# Genomic Programming of Human Neonatal Dendritic Cells in Congenital Systemic and *In Vitro* Cytomegalovirus Infection Reveal Plastic and Robust Immune Pathway Biology Responses

**DOI:** 10.3389/fimmu.2017.01146

**Published:** 2017-09-25

**Authors:** Widad Dantoft, Pablo Martínez-Vicente, James Jafali, Lara Pérez-Martínez, Kim Martin, Konstantinos Kotzamanis, Marie Craigon, Manfred Auer, Neil T. Young, Paul Walsh, Arnaud Marchant, Ana Angulo, Thorsten Forster, Peter Ghazal

**Affiliations:** ^1^Division of Infection and Pathway Medicine, School of Biomedical Sciences, University of Edinburgh, Edinburgh, United Kingdom; ^2^Immunology Unit, Department of Biomedical Sciences, Medical School, University of Barcelona, Barcelona, Spain; ^3^Quantitative Proteomics, Institute of Molecular Biology, Mainz, Germany; ^4^Synexa Life Sciences, Cape Town, South Africa; ^5^SynthSys-Centre for Synthetic and Systems Biology, School of Engineering, University of Edinburgh, Edinburgh, United Kingdom; ^6^Division of Applied Medicine, Institute of Medical Sciences, University of Aberdeen, Aberdeen, United Kingdom; ^7^NSilico Life Science and Department of Computing, Institute of Technology, Cork, Ireland; ^8^Institute for Medical Immunology, Université Libre de Bruxelles, Charleroi, Belgium

**Keywords:** infection, virus, immunity, systems biology, transcriptomics, set-point, congenital abnormalities, homeostasis

## Abstract

Neonates and especially premature infants are highly susceptible to infection but still can have a remarkable resilience that is poorly understood. The view that neonates have an incomplete or deficient immune system is changing. Human neonatal studies are challenging, and elucidating host protective responses and underlying cognate pathway biology, in the context of viral infection in early life, remains to be fully explored. In both resource rich and poor settings, human cytomegalovirus (HCMV) is the most common cause of congenital infection. By using unbiased systems analyses of transcriptomic resources for HCMV neonatal infection, we find the systemic response of a preterm congenital HCMV infection, involves a focused IFN regulatory response associated with dendritic cells. Further analysis of transcriptional-programming of neonatal dendritic cells in response to HCMV infection in culture revealed an early dominant IFN-chemokine regulatory subnetworks, and at later times the plasticity of pathways implicated in cell-cycle control and lipid metabolism. Further, we identify previously unknown suppressed networks associated with infection, including a select group of GPCRs. Functional siRNA viral growth screen targeting 516-GPCRs and subsequent validation identified novel GPCR-dependent antiviral (ADORA1) and proviral (GPR146, RGS16, PTAFR, SCTR, GPR84, GPR85, NMUR2, FZ10, RDS, CCL17, and SORT1) roles. By contrast a gene family cluster of protocadherins is significantly differentially induced in neonatal cells, suggestive of possible immunomodulatory roles. Unexpectedly, programming responses of adult and neonatal dendritic cells, upon HCMV infection, demonstrated comparable quantitative and qualitative responses showing that functionally, neonatal dendritic cell are not overly compromised. However, a delay in responses of neonatal cells for IFN subnetworks in comparison with adult-derived cells are notable, suggestive of subtle plasticity differences. These findings support a set-point control mechanism rather than immaturity for explaining not only neonatal susceptibility but also resilience to infection. In summary, our findings show that neonatal HCMV infection leads to a highly plastic and functional robust programming of dendritic cells *in vivo* and *in vitro*. In comparison with adults, a minimal number of subtle quantitative and temporal differences may contribute to variability in host susceptibility and resilience, in a context dependent manner.

## Introduction

Infection remains a leading cause of sickness and death in early life. While there has been extensive work investigating bacterial infections, viral infections are far less studied. Although diagnosing bacterial sepsis is highly problematic, viral infections are more often atypical and present an even greater challenge notwithstanding fewer treatment options in the newborn period. Host control of viral infections requires both effective innate and adaptive immune responses. In an indirect way, it can be considered that certain viral infection outcomes can be useful as a pan marker for both innate and adaptive immune health. A natural candidate example of an opportunistic viral-marker for human immune health is Human Cytomegalovirus (HCMV), the most common human viral infection acquired *in utero*. In this context, key features of HCMV that make it a paradigmatic biomarker of immune health include ubiquity as a human pathogen, and especially for early life where HCMV is a leading member of the ToRCH complex of perinatal infections. However, most critically, infection of immune-intact individuals results in benign asymptomatic infection while in an immune compromised setting HCMV rapidly becomes a major clinical complication.

Symptomatic congenital HCMV causes multi-system diseases that may present with intrauterine growth retardation, hepatosplenomegaly, cholestasis, rash, thrombocytopenia and intracranial and ophthalmological abnormalities, and hearing loss (1, 2). Although HCMV infections can be effectively acquired *in utero* only a small percentage of newborns from primary maternal infections (~1–10%) will develop congenital disease (1). Notably, it has been recently argued that maternal immune responses to HCMV, against existing dogma, have poor predictive value to protection against congenital disease severity (3). However, the possible role of fetal immune responses are not considered as they are historically and presently viewed as redundant to maternal protection. Furthermore, the virus can also be efficiently transmitted to the neonate at parturition from contact with vaginal secretions or subsequently at the point of breast milk feeding. However, these neonatal infections, inclusive of premature infected infants, usually result in little or no clinical illness (4). A corollary from all these observations is that while there is an important clinical risk to HCMV infection in early life, *in utero* as well as for premature and full-term neonates, there is a level of resilience that is, *de facto*, an intrinsic functional marker of the underlying neonatal immunity.

The above viewpoint on the unappreciated resilience of immune health in early life raises questions about the defensibility of the long accepted view that neonates (especially preterm) simply have increased susceptibility to infection due to an immature immune system that is not yet functionally intact and which is inherently immune deficient. The immune maturity/deficiency argument has also been increasingly challenged in animal neonatal model studies of infection (5–7). More recent human studies of neonatal sepsis and infection (8, 9) and human fetal innate immunity (10) are beginning to point toward a regulatory mechanism [reviewed in Ref. (7, 11)]. Examples of regulatory mechanisms encompass the concept of layered immunity during fetal development (12) and more recently to the notion of an altered set-point, through negative feedback, in innate immune triggering of stimulatory immune check-point control pathways, rather than developmental immune deficiency during the neonatal period (9, 11). In this regard, myeloid dendritic cells represent a central innate immune cell governing an early reference level for set-point control, acting as both a measuring element and an effector. In response to viral infections these cells rapidly produce type I interferon (IFN) and other inflammatory mediators, playing a key role for driving protective adaptive immunity.

In this report, we have sought to computationally investigate rare existing data resources for determining signal host pathway biology responses underpinning neonatal HCMV infections We ask whether the neonatal pathway biology, at the host systemic and infected cell level, provide a test for the robustness of early-life immunity and whether new insights toward host susceptibility and resilience can be gleaned. The selected studies include a previous transcriptional profiling study of whole blood samples of “well” or infected neonates (samples taken at time of first clinical presentation in neonatal intensive care unit), comprising mainly bacterial confirmed infections with a few case examples of confirmed viral infection inclusive of symptomatic congenital HCMV (9). While it has been well established that there is systemically high levels of circulating IFN in congenital HCMV infection (13), to date, the Smith et al. (9) study provides the only known available data for evaluating a systemic pathway response presented in congenital HCMV infection and which has previously not been investigated. For the purpose of exploring the neonatal cellular response to HCMV infection, we further investigated a unique study by Renneson et al. (14) that previously reported on immune profiling of adult and cord-blood myeloid-derived dendritic cells (DCs) upon HCMV infection. Those studies involved transcriptomic profiling of DCs derived from neonatal cord-blood and adult blood in response to HCMV infection. However, the central conclusions drawn in the previous published work were primarily based from ELISA assay data for the differential IL-12 and type I IFN responses (14). While these specific differences in the immunomodulatory cytokine response to HCMV infection between neonatal cord-blood cells and adult blood cells have been highlighted, global systematic unbiased genomic pathway biology analyses have yet to be reported.

Here, we investigate the hypothesis that neonates mount highly flexible and robust host pathway response to HCMV infection.

## Results

### Case Investigation of Systemic Cell Immune Response to Congenital HCMV Infection

**Figure 1:**
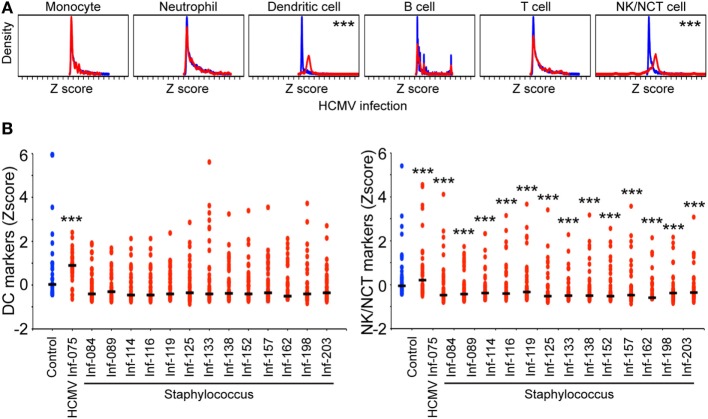
Activated expression of dendritic and natural killer (NK) cell markers upon congenital neonatal human cytomegalovirus (HCMV) infection. The expression of immune cell markers for monocytes, neutrophils, dendritic cell, B cell, T cells, and NK cells was investigated using transcriptomic analysis of whole blood isolated from a congenitally HCMV-infected neonate (*n* = 1, red) and uninfected neonatal controls (*n* = 35, blue). Using Wilcoxon Rank Sum test, adjusting for false discovery, a significantly activated expression profile was observed for dendritic cell (*p* < 1.3e−15) and NK/NCT cell (*p* < 1.8e−08) markers, but not for markers for monocytes, neutrophils, B, T cells. A *p*-value < 0.05 was considered significant, with *p < 0.05, **p < 0.01, and ***p < 0.001 **(A)**. The expression of immune cell markers for dendritic cells and NK/NCT cells in uninfected controls (*n* = 35, blue), one HCMV-infected neonate (*n* = 1, red) as in **(A)**, and 13 coagulase negative *Staphylococcus*-infected neonates (*n* = 13, red). Median expression is depicted as a black horizontal line and *p-*values < 0.05 were considered significant, with *p < 0.05, **p < 0.01, and ***p < 0.001 **(B)**.

To investigate the systemic cellular responses in neonates upon HCMV infection, the expression of monocyte, neutrophil, dendritic, B, T, and natural killer (NK)/NCT cell markers were analyzed in transcriptomic data, derived from neonatal blood obtained at the first clinical signs of a suspected infection, for a subsequently confirmed congenitally HCMV-infected female preterm infant (37 weeks gestational age). For comparison 13 preterm sepsis cases (5 females and 8 males from 27–39 weeks gestational age) with subsequently positive blood culture test result for causal *Staphylococcus* infection (clinical assessment for neonatal bacterial sepsis performed by two clinicians) (9). For these investigations of expression differences between the infected patient sample and the index control population (35 individual samples), the array data for each sample was *z*-score transformed prior to further analysis. For the HCMV-infected case (Inf-075) the distribution of markers for each individual subset of immune cells: monocytes (*n* = 211 markers), neutrophils (*n* = 164 markers), dendritic cells (*n* = 44 markers), B cells (*n* = 144 markers), T cells (*n* = 225 markers), and NK and non-conventional T cells (*n* = 59 markers) (co-inhibitory, co-stimulatory) was compared to the distribution in uninfected control samples using Wilcoxon Rank Sum tests (adjusted for false discovery) (Figure 1A; Table S2 in Supplementary Material). While changes in the distribution profile of markers for monocytes, neutrophils, B, and T cells are not observed, significant changes are detected for dendritic cells (*p* < 1.3e−15, adjusted) and for NK/NCT cells (*p* < 1.8e−08, adjusted).

**Figure 2:**
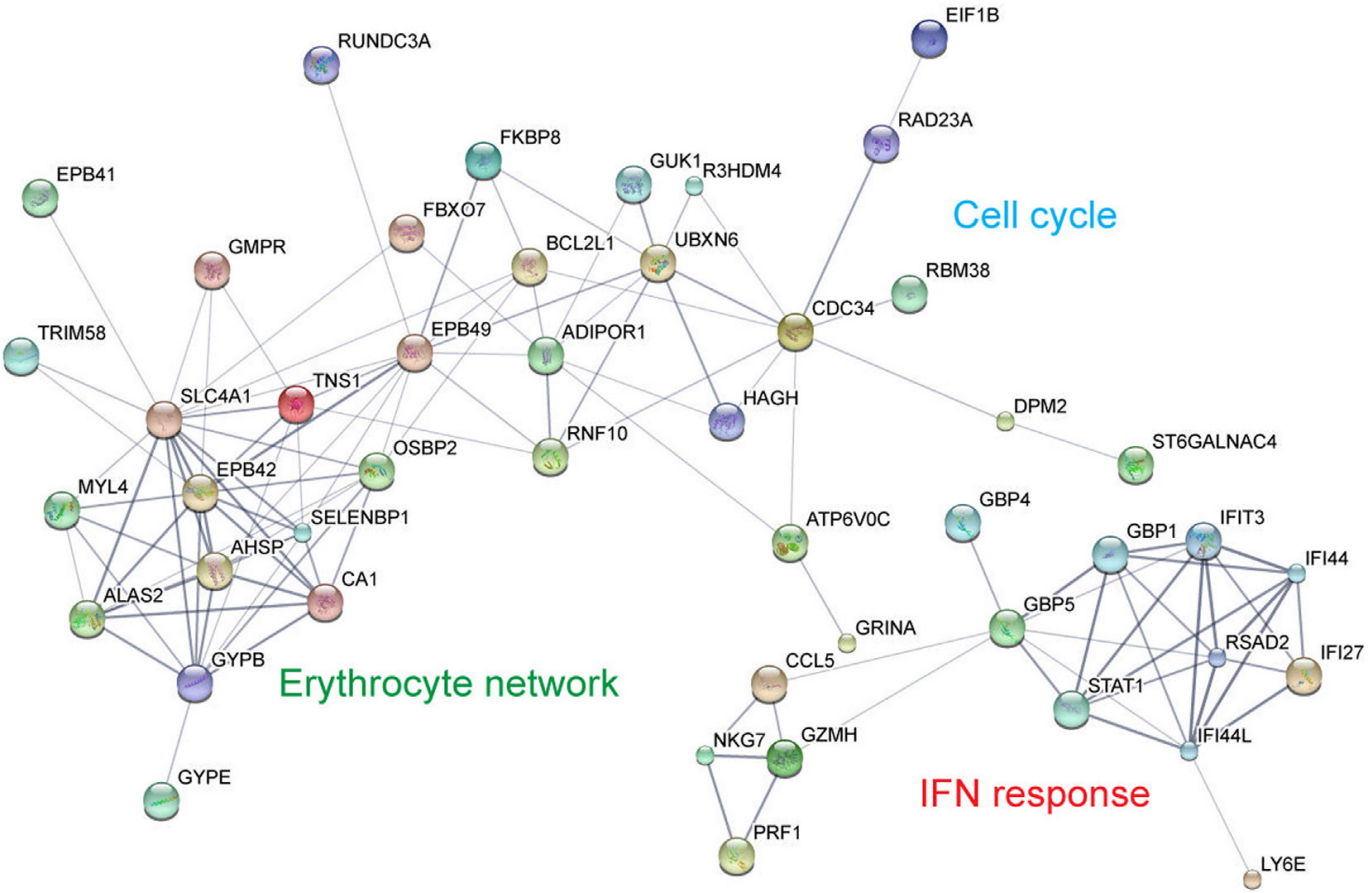
Differentially expressed genes upon congenital neonatal human cytomegalovirus (HCMV) infection form distinct subnetworks. STRING Network analysis was performed on the 114 genes with the highest significant differential expression (absolute log_2_FC ≥ 1.58, adjusted *p* < 0.05) upon congenital neonatal HCMV infection (Inf-075). Network analysis reveals the presence of two interconnected subnetworks comprising erythrocyte-specific genes (erythrocyte network), cell metabolic genes, and cell-cycle genes (cell cycle), as well as a distinct and separate subnetwork of IFN-induced genes with antiviral activity (IFN response).

To evaluate the global effects of congenital HCMV infection on the systemic host response, biological network relationship analysis was performed on the 114 genes with highest differential expression (fold change ≥3). Network analysis revealed the presence of two interconnected subnetworks comprising erythrocyte-specific genes (erythrocyte network) that may be confounded by development, and cell metabolic genes and cell-cycle genes (cell cycle), as well as a distinct subnetwork (IFN response) composed of IFN-induced genes involved in antiviral host responses, apoptosis, and the formation of the NLRP3 inflammasome that promotes the maturation of the inflammatory cytokines IL1β and IL18 (15) (Figure 2).

It is important to note that these findings are limited to the profile of congenital cytomegalovirus disease of an individual patient response and will require larger population studies to draw any general conclusions. However, computational pathway statistical approaches can be used to explore this case in comparison with controls and clearly highlights, in the case of patient Inf-075 congenital cytomegalovirus infection, a key role for myeloid dendritic, but not monocyte or neutrophil cells, and a dominant association with a systemic IFN antiviral subnetwork response. Further, we note that in comparison with healthy controls, elevated distribution levels of NK/NCT cell markers are also present in the systemic response of congenital HCMV. These responses are in marked contrast to individual bacterial (*Staphylococcus*) infection responses, which show no significant alteration in the distribution of dendritic cell markers in any of the cases examined, in contrast showing a slight trend toward an overall suppression, in particular, for patients Inf-089, 116, and 162, respectively (Figure 1B). Notably, the selective increase density distribution for NK/NCT cell markers in the HCMV case is in contrast to their significant suppression in the staphylococcal infection cases (Figure 1B), highlighting the flexibility of the innate immune systemic response in neonates.

### IFN Responses Are Dominant Early Signatures of HCMV Infection of Cord and Adult DCs Followed, at Late Times, by a Limited Differential Lipid Metabolic Pathway Response

The DCs not only act as key innate immune set-point control cells for triggering the adaptive arm but are also targets of viral infection. This further motivated us to investigate the pathway biology response of neonatal dendritic cells to HCMV challenge. For this purpose, we proceeded to investigate transcriptomic data (Human U 133 Plus 2.0 genome array) of infected monocyte-derived dendritic cells (DCs) from neonatal cord-blood over time, previously reported by Renneson et al (14). Here, neonatal (cord) and adult-derived monocyte-derived dendritic cells (DCs) were either mock treated or infected with HCMV and RNA harvested for genome-wide array analysis at 6 and 16 h postinfection. After normalization and filtering of the 54,676 gene probes, hybridized with labeled DCs RNA on the Human U 133 Plus 2.0 genome array platform, 1,862 probes were considered significantly differentially expressed from mock-infected controls with an absolute expression value of log_2_FC ≥ 1, and an adjusted *p*-value < 0.05 after false discovery correction.

**Figure 3:**
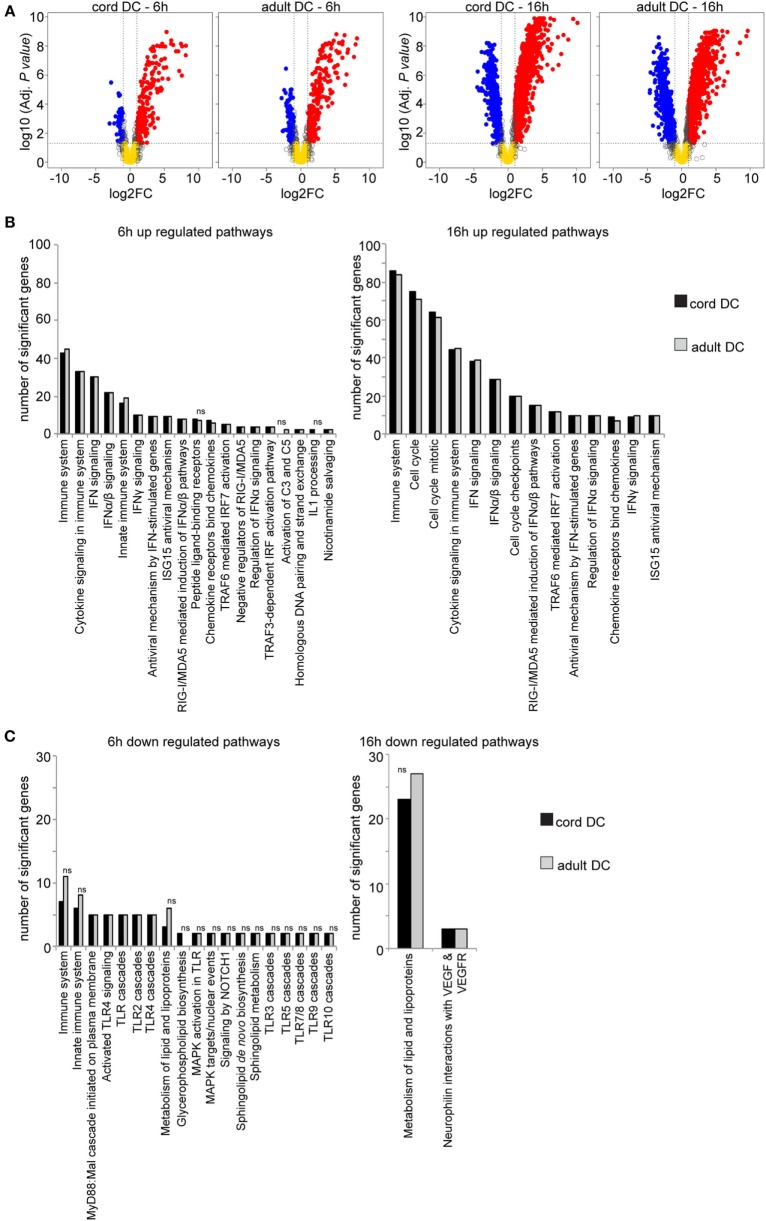
HCMV-infected cord-derived DCs display an activated antiviral pathway response dominated by IFN-induced cytokine signaling, increased cell-cycle activation, and a suppression of lipid metabolism. Volcano plots showing the genome-wide expression profile in cord and adult-derived DCs upon 6 and 16 h of HCMV infection **(A)**. Graphs visualizing the over-represented pathways (ORA), and the number of significantly expressed genes grouped within each ORA, present among the significantly upregulated genes **(B)** and significantly downregulated genes **(C)** at 6 and 16 h postinfection in cord (black bars) and adult (gray bars) DCs **(B,C)**. The genes grouped in Immune system and Metabolism of lipid and lipoproteins are further sub-categorized into several immune system and lipid metabolism-related pathways. Pathways not identified in one of the two datasets (but present in the other), or pathways with non-significant corrected *p*-values (*p* > 0.05), are marked with “ns”.

After 6 h postinfection, 272 genes were significantly differentially expressed in cord DCs (compared to mock-infected DCs), exhibiting a strongly activated profile (Figure 3A). Out of these, 222 were significantly upregulated and 50 genes significantly downregulated, a greater number than previously reported. Note that this difference depends on the chosen parameters for analysis (data pre-processing algorithms, non-specific filtering, type of test and multiple-testing adjustment used, as well as level of fold change considered). For comparative purposes, 6 h post HCMV infection of adult DCs, 316 genes are significantly differentially expressed, exhibiting a strongly activated profile that is highly similar to that of infected cord DC. Out of these 316 genes, 221 were significantly upregulated and 95 genes were significantly downregulated.

Figure 3A further shows that at 16 h post HCMV infection, a greater number of differentially regulated genes are further detected in adult and cord DCs. In the case of cord DCs, an increased number of 1,474 genes were significantly expressed, with 1,124 genes upregulated and 350 genes downregulated. A similar number of differentially expressed genes were observed in adult DC (1,402), out of which 1,031 genes were upregulated and 371 were downregulated.

In HCMV-infected cord DCs, consistent with Renneson et al., an over representation of immune system pathways was observed among the upregulated genes (14), with a focus on cytokine signaling and type I IFN response (*IFNA1* and *IFNB1*, IFN signaling pathway) mediated by the RIG/MDA5 signaling activation as well as type III IFNs (Figure 3B). Many IFN-induced/stimulated genes with reported antiviral activity, including *IFI6, IFI35, IFIT1, IFIT2, IFIT3, IRF7, ISG15*, and *ISG20*, were found to be significantly upregulated after 6 h of infection, as well as an induced expression of various type I IFNs (Table 1), cytokines (Table 2), and chemokines (Table 3). Phenotypically the overall cytokine response has a hallmark for a strongly activated pro-inflammatory response. We further note that apart from *IRF7*, no other interferon regulatory factors (IRFs) were detected. Notable, interferon response factor 2 binding protein 2 (*IRF2BP2*) that in an IRF2-dependent manner functions as a transcriptional co-repressor was downregulated. Strikingly, these responses are mirrored in the 6 h post infected adult DCs.

**Table 1:** Fold-change expression of interferon genes in HCMV-infected cord and adult DCs.

Gene	Cord 6 h	Cord 16 h	Adult 6 h	Adult 16 h
IFNA1	17.88	271.56	37.32	366.51
IFNA2	nd	11.96	nd	12.65
IFNA4	nd	11.55	nd	32.99
IFNA5	nd	8.71	nd	12.13
IFNA6	nd	nd	nd	nd
IFNA7	nd	6.33	nd	12.08
IFNA8	nd	27.34	nd	44.33
IFNA10	nd	2.91	nd	4.09
IFNA13	nd	19.01	4.56	33.32
IFNA14	nd	10.53	nd	18.06
IFNA16	nd	11.35	nd	22.92
IFNA21	nd	3.51	nd	4.75
IFNB1	18.44	9.12	50.34	20.44
IFNE	nd	nd	nd	nd
IFNG	nd	nd	nd	9.53
IFNL1 (IL29)	nd	nd	nd	nd
IFNL2 (IL28A)	nd	nd	nd	nd
IFNW1	nd	4.44	nd	5.00

*nd, not detected*.

**Table 2:** Fold-change expression of cytokine genes in HCMV-infected cord and adult DCs.

Gene	Cord 6 h	Cord 16 h	Adult 6 h	Adult 16 h
IL1A	2.76	nd	nd	−2.59
IL1B	7.06	2.14	3.46	nd
IL2	nd	nd	nd	nd
IL3	nd	nd	nd	nd
IL4	nd	nd	nd	nd
IL5	nd	nd	nd	nd
IL6	nd	nd	nd	nd
IL7	3.10	3.15	4.02	2.55
IL9	nd	nd	nd	nd
IL10	nd	nd	nd	nd
IL11	nd	nd	nd	nd
IL12B	nd	nd	nd	nd
IL13	nd	nd	nd	nd
IL15	2.75	nd	6.16	2.26
IL16	−2.63	−5.75	−2.78	−7.96
IL17A	nd	nd	nd	nd
IL18	nd	nd	nd	nd
IL24	nd	nd	nd	nd
IL27	nd	nd	nd	nd
IL32	nd	nd	nd	nd
IL33	nd	nd	nd	nd

*nd, not detected*.

**Table 3:** Fold-change expression of chemokine genes in HCMV-infected cord and adult DCs.

Systematic name	Common name	Cord 6 h	Cord 16 h	Adult 6 h	Adult 16 h
CCL1	I-309	nd	nd	nd	nd
CCL2	MCP-1	9.18	6.67	5.62	3.28
CCL3	MIP-1α	nd	nd	nd	nd
CCL4	MIP-1β	9.96	5.58	3.74	nd
CCL5	RANTES	4.69	19.80	7.78	27.86
CCL7	MCP-3	4.59	12.11	5.55	7.54
CCL8	MCP-2	33.73	54.62	43.33	60.70
CCL11	Eotaxin	nd	nd	nd	nd
CCL13	BCA-1	nd	nd	nd	nd
CCL17	TARC	nd	nd	nd	nd
CCL19	ELC	nd	2.14	nd	nd
CCL20	MIP-3α	4.83	8.92	nd	6.51
CCL21	SLC	nd	nd	nd	nd
CCL22	MDC	nd	nd	nd	nd
CCL23	MIP3	nd	nd	nd	nd
CCL25	TECK	nd	nd	nd	nd
CCL27	DTACK	nd	nd	nd	nd
CCL28	MEC	nd	2.00	nd	nd
CX3CL1	Fractaline/neurotactin	nd	nd	nd	nd
CXCL1	GRO-α	nd	nd	nd	nd
CXCL2	GRO-β	nd	nd	nd	nd
CXCL3	GRO-γ	nd	nd	nd	nd
CXCL5	ENA78	nd	nd	nd	nd
CXCL6	GCP-2	nd	nd	nd	nd
CXCL7	NAP-2	nd	nd	nd	nd
CXCL8	IL8	5.52	2.65	4.39	nd
CXCL9	MIG	10.18	90.94	23.80	75.06
CXCL10	IP-10	280.55	438.22	216.16	288.74
CXCL11	I-TAC	286.21	1,113.54	253.76	691.84
CXCL12	SDF-1	nd	nd	nd	nd
CXCL13	BCA-1	nd	nd	nd	nd
CXCL14	BRAK	nd	nd	nd	nd
CXCL16	–	nd	nd	nd	nd
XCL1	Lymphotactin	nd	nd	nd	nd

*nd, not detected*.

The number of upregulated genes in these pathways increased with the progression of infection (from 6 to 16 h of infection) for both neonates and adults (Figure 3B, right panel), suggesting that an increased innate immune function dominates a large portion of the activated profile observed at 16 h of infection (Figure 3A). In addition to an increased number of activated genes involved in immune system processes, genes involved in maintaining the cell cycle were observed at 16 h of infection and the overall robustness of response is similar between neonate and adult (Figure 3B). This outcome was not expected and is suggestive of plasticity between neonatal and adult DCs responses.

Notably, *TLR7*, previously shown to recognize single-stranded RNA from HIV, HCV, and HCMV in other systems (16–18), was the only *TLR* gene found to be upregulated in the infected cord-derived DCs. With an initially unchanged expression at 6 h of infection, its expression was significantly upregulated at 16 h of infection, suggesting a delayed enhancement of this TLR gene during infection. *TLR4, TLR6*, and *TLR8* all demonstrated a downregulated expression in infected cord DCs. In this connection, Smith et al. showed an upregulation of *TLR4*, 5, 7, and 8 in HCMV-infected macrophages, while *TLR1, TLR2, TLR3*, and *TLR5* were unchanged (17). Genes categorized as belonging to immune system pathways (including *TLR6, IFNGR1*, and *FOS*) as well as TLR cascades (*CD36, CTSB, FOS, JUN, TLR4, TLR6*, and *TLR8*), with reported partial immunoregulatory roles, were also present among the downregulated genes (Figure 3C). Notably, *CD36*, a multifunctional glycoprotein, has been shown to function as a co-receptor for the TLR4:TLR6 heterodimer, promoting inflammation in monocytes/macrophages (19).

As the infection progressed, an increased number of downregulated genes was also similarly detected for neonatal and adult cells, with the exception of genes associated with the glycerophospholipid biosynthesis pathway that is exclusively observed at early times for cord infected cells (Figure 3C). There is a notable shift from a modest number of genes at the 6 h time point categorized as belonging to the sphingolipid *de novo* biosynthesis pathway (2 in cord and adult cells, respectively) and metabolism of lipid and lipoprotein (3 and 6 in cord and adult, respectively), to a clear increase in the number of downregulated genes (23 and 27 in cord and adult, respectively) involved in metabolism of lipids and lipoproteins (including genes involved in sphingolipid *de novo* biosynthesis and triglyceride biosynthesis) (Figure 3C; Table 4). Moreover, the two over-represented pathways nucleotine-like (purinergic) receptors and signaling by NOTCH1, at 6 h of infection, are not significantly over-represented at 16 h. Instead a small number of genes are grouped as belonging to neurophilin interactions with VEGF and VEGFR are overrepresented at 16 h. Unlike lipid metabolism, the expression of genes involved in glycolysis/gluconeogenesis and the citrate cycle (TCA) were moderately changed at 6 and 16 h of infection (Tables 5 and 7). In particular, only a few glycolytic/glucanogenic genes exhibited an infection-induced change in expression (*PGM2L1, LDHAL6B, ENO3, ALDH8A1*, and *ALDH18A1*). While *LDHAL6B* was initially upregulated at 6 h of infection, its expression was undetectable later in the infection (16 h). *PGM2L1, ALDH8A1*, and *ALDHL18A1* on the other hand all displayed a delayed response and were only significantly upregulated by 16 h of infection, while *ENO3* exhibited a downregulated response at 16 h.

**Table 4:** Fold-change expression of genes involved in metabolism of lipids and lipoproteins in HCMV-infected cord and adult DCs.

Gene	Cord 6 h	Cord 16 h	Adult 6 h	Adult 16 h	Step
ABCA1	nd	−2.02	nd	nd	Efflux of cholesterol and phospholipids
ABCB4	nd	nd	nd	−4.38	Phosphatidylcholine transporter
ABCC3	nd	−5.96	−2.75	−7.49	Not determined
ACOX2	nd	−3.96	nd	−3.42	Degradation of long branched fatty acids (FA)
ACSL5	nd	−3.57	nd	−4.47	Converts free long-chain fatty acid (LCFA )> FA-CoA esters, FA degradation *via* beta-oxidation
AKR1C3	nd	−4.71	nd	−18.28	Aldehydes and ketones > corresponding alcohols
ALOX5	nd	−3.41	nd	−2.82	Catalyzes first step in leukotriene biosynthesis, important for inflammatory response
ASAH2	3.01	8.92	3.38	5.69	Ceramide > sphingosine
CD36	−2.02	−2.77	−3.67	−4.66	Binds LDL among others, supports inflammatory response
CROT	nd	5.96	nd	3.66	4,8-dimethylnonanoyl-CoA > carnitine ester
CYP1A1	4.82	nd	3.80	nd	Substrate unknown
CYP1B1	nd	−2.95	nd	−5.44	Estradiol > 4-OH-estradiol or 2-OH-estradiol
CYP27A1	nd	−4.71	nd	−6.55	First step in the oxidation of side chains of sterol intermediates
CYP27B1	nd	−4.12	−2.53	−4.02	25(OH)D3 > 1,25-(OH)_2_D_3_ (Calcitrol)
CYP2U1	nd	7.99	nd	7.06	LCFA > biologically active epoxides
CYP7B1	nd	4.10	nd	3.01	25-HC > 7-alpha,25-OHC
ELOVL4	nd	9.67	nd	7.96	C*_n_*COOH > C*_n_* + 2COOH (*n* > 16)
ELOVL6	nd	2.96	nd	2.58	C*_n_*COOH > C*_n_* + 2COOH (*n* > 16)
ELOVL7	2.20	5.93	nd	6.62	C*_n_*COOH > C*_n_* + 2COOH (*n* > 16)
FABP4	nd	−3.69	nd	−4.97	Lipid transport protein
G0S2	nd	nd	nd	−5.68	Inhibits the rate-limiting lipolytic enzyme ATGL
GPAT3	nd	−5.81	nd	−17.02	Lysophosphatic acid (LPA) > phosphatic acid (PA)
glycerol-3-phosphate dehydrogenase 1	−2.72	−3.81	nd	−3.26	Dihydroxyacetone phosphate + NADH > glycerol-3-P + NAD+
INSIG1	8.44	nd	4.65	nd	Inhibits SREBP activation, binds SCAP and HMG-CoA
LCLAT1	nd	5.55	nd	5.81	LPA > PA
LDLR	4.41	nd	nd	−2.29	Binds cholesterol-carrying LDL, promotes endocytosis
ME1	nd	nd	nd	−3.59	Generates NADPH for FA biosynthesis
MED30	nd	4.02	nd	3.42	May play a role in cardiovascular disease
PEX11A	nd	2.50	nd	3.10	Promotes membrane protrusion and elongation on the peroxisomal surface
PIK3C2A	nd	2.05	nd	nd	Functions in clathrin-coated endocytic vesicle formation and distribution
PIK3CG	nd	−3.14	nd	−6.06	PtdIns(4,5)P2 > PIP3, immune activator
PIK3R3	nd	4.67	2.67	4.10	PtdIns > PIP
PIP5K1B	nd	4.39	nd	6.03	Participates in the biosynthesis of phosphatidylinositol 4,5-bisphosphate
PLA2G12A	nd	4.25	nd	4.12	Membrane phospholipids > Free FAs + lysophospholipids
PLA2G4C	nd	3.84	nd	nd	Hydrolyzes phospholipids > FA + other lipophilic molecules
PLA2G5	nd	−3.96	nd	nd	Membrane phospholipids > FFA + lysophospholipids
PNPLA3	−2.04	−5.01	−3.05	−5.49	Mediates triacylglycerol hydrolysis
PLPP3	−2.52	−5.68	−3.05	−7.62	PA > DG
PPARG	nd	−5.59	nd	−9.54	Forms dimer with RXR to activate gene expression
PPM1L	nd	−2.38	nd	−2.78	Dephosphorylates MAP3K7 and MAP3K5
PRKAA2	nd	nd	nd	2.42	Catalytic subunit of AMPK
PRKACB	nd	−3.49	nd	−4.38	cAMP dependent PKA subunit
PTGS2	22.61	21.47	19.45	14.24	Arachidonate > prostaglandin H2
RORA	nd	22.20	nd	17.10	Mediates cell binding, signaling, and cytoskeletal organization
SEC24A	nd	2.91	nd	2.01	Component of COPII-coated vesicle
SGMS2	2.15	5.24	nd	3.32	Ceramide >< sphingomyelin
SLC27A2	nd	6.63	nd	7.53	LCFA > fatty acyl-CoA esters
SPTLC2	2.72	3.63	3.76	3.24	l-serine + palmitoyl-CoA > 3-oxo-sphinganine
SQLE	nd	nd	nd	−2.19	Squalene > 2,3-oxidosqualene
TBXAS1	nd	−4.58	nd	−5.38	Prostaglandin H2 > thromboxane A2
UGCG	nd	nd	nd	nd	1-ceramide > 4GLCβ1
VAPA	−2.28	−3.10	−3.27	−4.53	Binds OSBPL3, stimulates RRAS signaling

*nd, not detected; >, conversion*.

**Table 5:** Fold-change expression of genes involved in triglyceride biosynthesis in HCMV-infected cord and adult DCs.

Gene	Cord 6 h	Cord 16 h	Adult 6 h	Adult 16 h	Step
ACSL5	nd	−3.57	nd	−4.47	FAs > Acyl-CoA
ELOVL4	nd	9.67	nd	7.96	C*_n_*COOH > C*_n_* + 2COOH (*n* > 16)
ELOVL6	nd	2.96	nd	2.58	C*_n_*COOH > C*_n_* + 2COOH (*n* > 16)
ELOVL7	2.20	5.93	nd	6.62	C*_n_*COOH > C*_n_* + 2COOH (*n* > 16)
GPAT3	nd	−5.81	nd	−17.02	LPA > PA
glycerol-3-phosphate dehydrogenase 1	−2.72	−3.81	nd	−3.26	Dihydroxyacetone-P > glycerol-3-P
LCLAT1	nd	5.55	nd	5.81	Lysophosphatic acid > PA

*nd, not detected, >, conversion*.

The number of downregulated genes involved in metabolism and transport of lipids and lipoproteins increased from 4 [glycerol-3-phosphate dehydrogenase 1 (*GPD1*), *PLPP3 (PPAP2B), PNPLA3*, and *VAPA]* at 6 h to 22 genes at 16 h (*ABCA1, ABCC3, ACOX2, ACSL5, AKR1C3, ALOX5, CD36, CYP1B1, CYP27A1, CYP27B1, FABP4, GPAT3, GPD1, PIK3CG, PLA2G5, PNPLA3, PLPP3, PPARG, PPM1L, PRKACB, TBXAS1*, and *VAPA*). Further investigation of genes involved in metabolism of lipids and lipoproteins revealed an activation of *ASAH2, CROT, CYP1A1, CYP2U1, CYP7B1, ELOVL4, ELOVL6, ELOVL7, INSIG1, LCLAT1, LDLR, MED30, PEX11A, PIK3C2A, PIK3R3, PIP5K1B, PLA2G12A, PLA2G4C, PTGS2, RORA, SEC24A, SGMS2, SLC27A2*, and *SPTLC2* at 6 or 16 h of infection (Table 4).

Further sub-categorization using REACTOME (20, 21) revealed pathways for triglyceride biosynthesis (*ACSL5, GPAT3*, and *GPD1*) and sphingolipid *de novo* biosynthesis (*PLPP3, PPM1L*, and *VAPA*) (Tables 5 and 6). However, further analysis revealed activation of several other pathway members; including *ELOVL4, ELOVL6, ELOVL7*, and *LCLAT1* for triglyceride biosynthesis (Table 5), and *INSIG1, LDLR, CD36*, and *CYP27A1* for the sterol metabolic network (Table 4), *SGMS2* and *SPTLC2* for sphingolipid *de novo* biosynthesis, respectively (Table 6). While *ELOVL7* was activated already at 6 h of infection, both *ELOVL4* and *ELOVL6* displayed a delayed response (activated at 16 h of infection). Elongation of very long-chain fatty acids (ELOVL) 4, 6, and 7 are membrane bound proteins that all catalyze the first rate-limiting step in the elongation of very long fatty acids (FAs) (22–24), required for the biosynthesis of lipids, sphingolipids, and eicosanoids.

**Table 6:** Fold-change expression of genes involved in sphingolipid *de novo* biosynthesis in HCMV-infected cord and adult DCs.

Gene	Cord 6 h	Cord 16 h	Adult 6 h	Adult 16 h	Step
PPLP3	nd	−2.38	nd	−2.78	Phosphatic acid > Diacylglycerol
PPM1L	2.15	5.24	nd	3.32	Dephosphorylates MAP3K7 and MAP3K5
SGMS2	2.72	3.63	3.76	3.24	Ceramide >< sphingomyelin
SPTLC2	−2.28	−3.10	−3.27	−4.53	l-serine + palmitoyl-CoA > 3-oxo-sphinganine
VAPA	nd	−2.38	nd	−2.78	Binds OSBPL3, stimulates RRAS signaling

*nd, not detected, >, conversion*.

**Table 7:** Fold-change expression of genes involved in glycolysis/glucanogenic in HCMV-infected cord and adult DCs.

Gene	Cord 6 h	Cord 16 h	Adult 6 h	Adult 16 h	Step
ALDH18A1	nd	6.36	nd	9.26	Glutamate > glutamate 5-semialdehyde
ALDH8A1	nd	2.87	nd	3.96	9-cis-retinal > 9-cis-retinoic acid
ENO3	nd	−2.45	nd	nd	2-phosphglycerate > phosphoenolpyrovate
LDHAL6B	4.54	nd	4.82	nd	Paralog of LDHAL6A
PGM2L1	nd	3.50	nd	2.57	Synthesizes glucose-1,6-bisphosphate and glucose-1,5-bisphosphate

*nd, not detected; >, conversion*.

HCMV has been shown to not only extensively alter the host cell’s metabolism, including inducing FA metabolism (25), but also alter vesicle membranes. It is thus assumed that HCMV manipulates the host cell’s lipid metabolism to facilitate virion assembly and/or to use FAs as structural components. Interesting, *ELOVL7* is known to be inducible by HCMV in infected human foreskin fibroblasts (26). Notably, Koyuncu et al. (27) showed that inhibition of FA metabolism affects not only to the formation of new virions but also infectivity of the newly produced virions (27). Nevertheless, it is clear that HCMV infected host cells synthesize saturated very long-chain fatty acids (VLCFAs), which appear to be incorporated in its virion lipid membrane (27). Furthermore, ELOVL7 is the one of the ELOVLs associated with the synthesis of elongation of very long FAs (24), revealing an association between metabolism and HCMV infection. However, it is worth noting that these responses are also likely to be important for immunomodulatory functions.

Conversely, *LCLAT1*, which converts lysophosphatic acid (LPA) to phosphatic acid (PA), remained unchanged from mock-infected cells during early infection but was subsequently upregulated as the need for lipids increased. This is possibly required for the maintenance of the cell expansion, as indicated by the upregulation of genes involved in cell division and cycle progression (Figure 3B) and may represent a viral high-jacking of lipid metabolism to build virions. Intriguingly, the expression of *GPAT3* that encodes an enzyme that also converts LPA to PA, remained unchanged at 6 h but are subsequently downregulated as the infection proceeded, displaying an opposing expression pattern from *LCLAT1* during late infection. *GPD1*, encoding GPD1 was downregulated in cord DCs already from 6 h of infection and stayed downregulated as the infection proceeded, while in adults it remained unchanged until 16 h, at which it was downregulated. GDP1 has been shown to convert dihydroxyacetone phosphate and NADH, from the glycolysis pathway, to glycerol-3-P and NAD+, which feeds into triglyceride biosynthesis (28). Finally, *ACSL5* encoding ACSL5, which converts long-chain fatty acids to fatty acyl-CoA esters, was downregulated in the HCMV-infected DCs.

Taken together, these results suggest that the initial antiviral innate immune response, to HCMV infection in cord-blood and adult peripheral derived DCs, is dominated by a strong IFN and pro-inflammatory cytokine signaling, and chemokine receptor binding, centered on the activation of ISG15 (Figure 3). As the infection progresses, a heightened innate immune gene activation and increased cell-cycle activation and metabolic changes are observed. Overall, there is a striking similarity of the responses between neonatal and adult-derived DCs to HCMV infection, with only minor quantitative and qualitative differences exhibited. These results strongly suggest that neonatal dendritic cells have the capacity to produce a robust and flexible innate immune response to viral infection.

### Neonatal Molecular Pathways Develop Robust Pro-Inflammatory Subnetwork Responses Upon Infection

**Figure 4:**
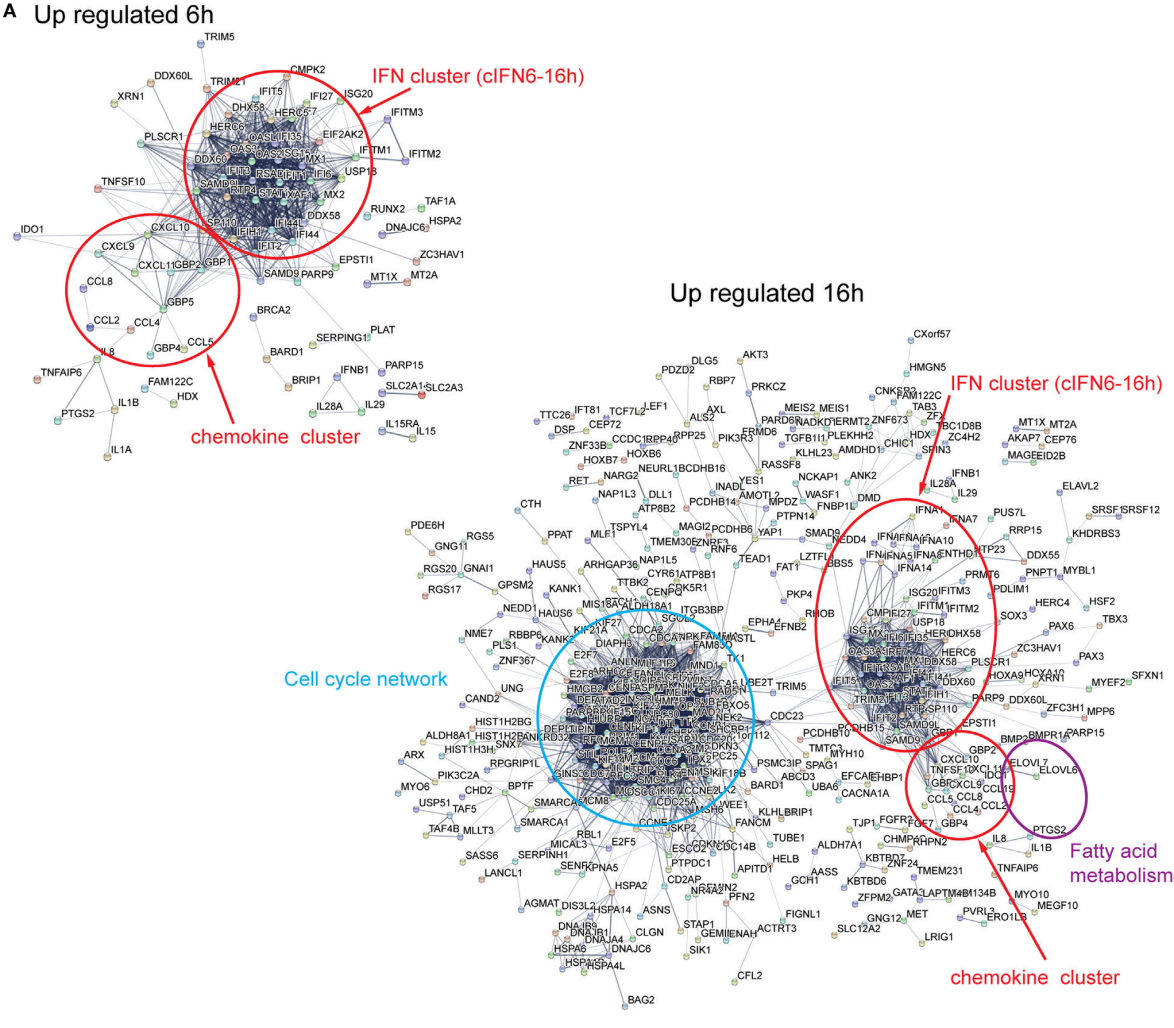

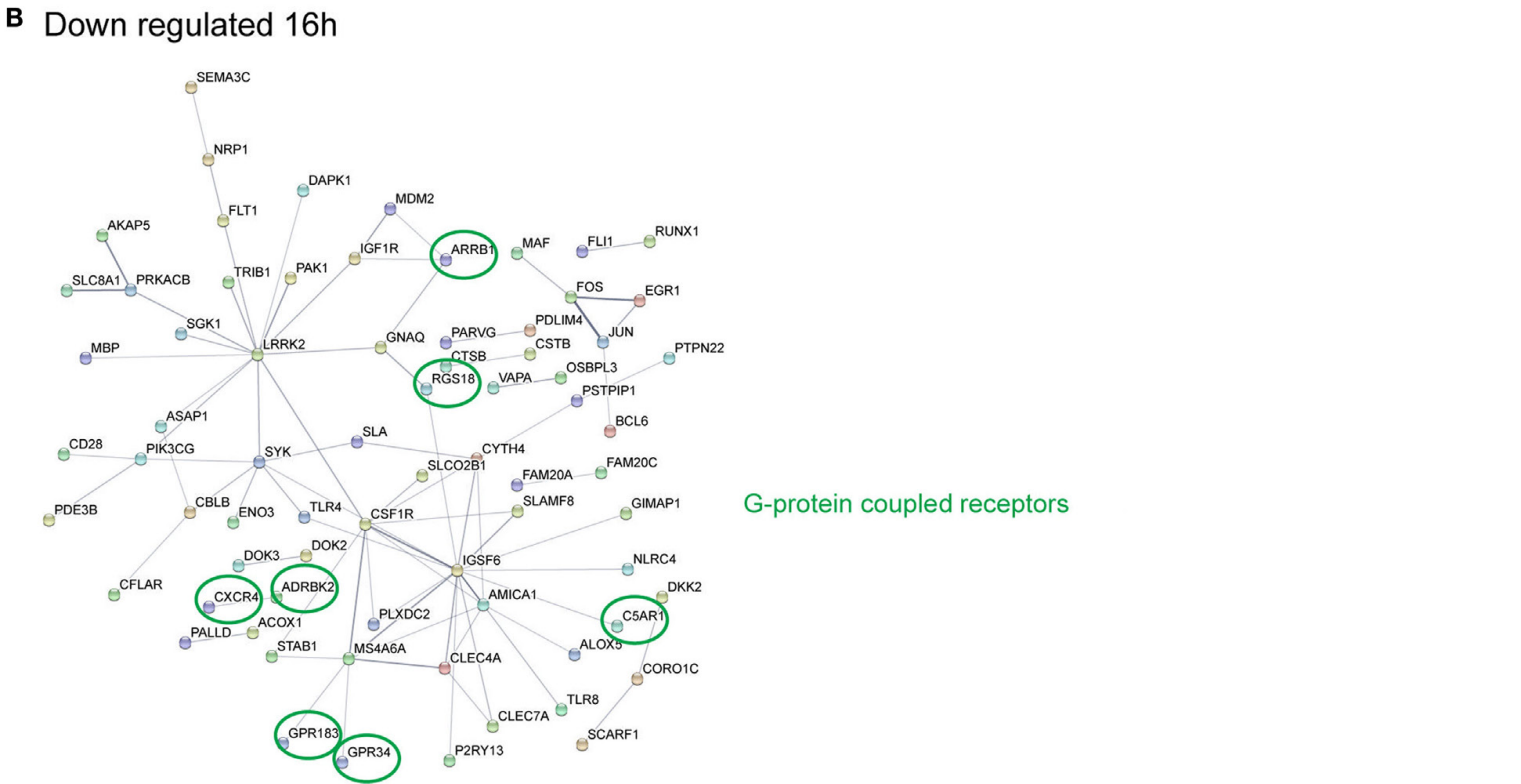
Differentially expressed genes in HCMV-infected cord DCs form distinct subnetworks. STRING Network analysis of up- and downregulated genes in cord DCs, form at 6 and 16 h of infection distinct subnetworks **(A,B)**. Among the upregulated genes, after both 6 and 16 h of infection, two immune-related subnetworks are identified [IFN cluster (cIFN6-16h) and chemokine cluster] and at 16 h, specifically, a network of cell-cycle-related genes (cell-cycle network) and a network of fatty acid (FA) metabolism genes (FA metabolism) are identified **(A)**. Among the downregulated genes several G-protein coupled genes (encircled) are identified at 16 h of infection **(B)**. The identified members of each network can be found in Table S1 in Supplementary Material.

**Figure 5:**
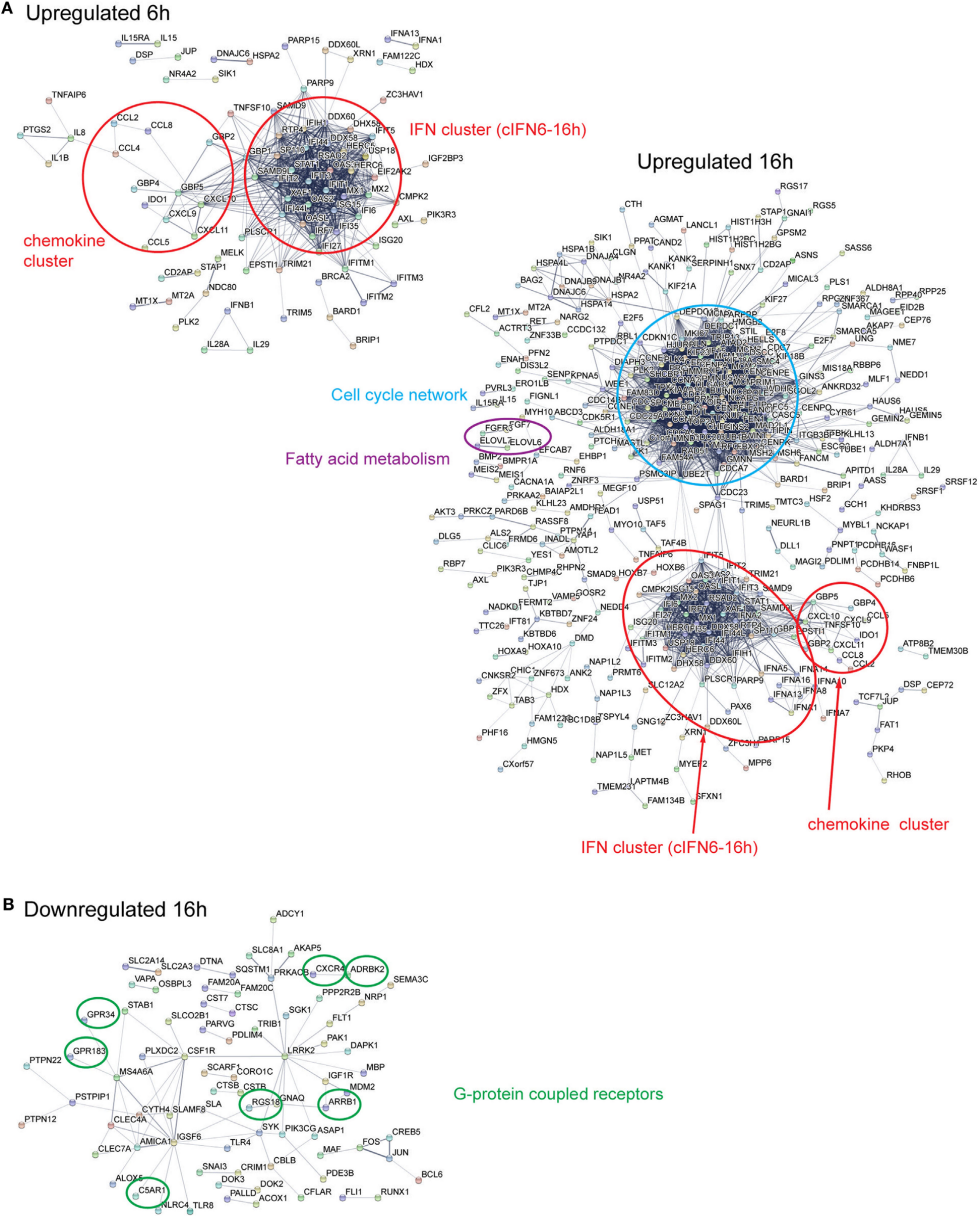
Differentially expressed genes in HCMV-infected adult DCs form distinct subnetworks similar to those in neonatal cord DCs. STRING Network analysis of up- and downregulated genes in adult DCs, form at 6 and 16 h of infection distinct subnetworks **(A,B)**. Among the upregulated genes, after both 6 and 16 h of infection, two immune-related subnetworks are identified [IFN cluster (cIFN6-16h) and chemokine cluster] and at 16 h a network, specifically, of cell-cycle-related genes (cell-cycle network) and network of fatty acid (FA) metabolism genes (FA metabolism) are identified **(A)**. Among the downregulated genes several G-protein coupled genes (encircled) are identified at 16 h of infection **(B)**. The identified members of each network can be found in Table S1 in Supplementary Material.

To further corroborate and explore the response to HCMV infection of DC we undertook an integrative STRING and InnateDB analysis to define subnetworks of genes regulated in the course of HCMV infection of neonatal cord (Figure 4) and adult (Figure 5) derived DCs.

STRING network analysis of the neonatal upregulated genes revealed, at 6 and 16 h, the formation of distinct subnetworks of IFN-responsive genes (IFN cluster, cIFN6-16h) and chemokines (chemokine cluster) that as the infection proceeded increased in complexity (Figure 4A; Table S1 in Supplementary Material). At 6 h of infection, the IFN repertoire was limited to *IFNA1* and *IFNB1* but at 16 h it had increased sevenfold in number (*IFNA1, IFNA2, IFNA4, IFNA5, IFNA7, IFNA8, IFNA10, IFNA13, IFNA14, IFNA16, IFNA21, IFNB1*, and *IFNW1*) (Table 1). Conversely, the expression of *IFNAR1* and *IFNGR1* was either unchanged by infection or downregulated with time, respectively, indicative of plasticity in regulating the type I IFN response and a disengagement of the type II IFN response. Moreover, two IFN-stimulated genes (ISGs), *ISG15*, and *ISG20*, as well as genes involved in ISG15 antiviral mechanism (*DDX58, IFIT1, EIF2AK2, MX1, MX2, HERC5*, and *STAT1*) were found be induced in the infected cord DCs at both time points after infection, indicating a strict regulation of cellular antiviral responses with counter balance through the induction of USP18 for both neonatal cord and adult cells (Table S1 in Supplementary Material).

Interleukin gene activation was limited to *IL1A, IL1B, IL7*, and *IL15* at 6 h, followed by a downregulated expression of all genes, except *IL7*, at 16 h of infection. Pro-inflammatory chemokine expression of *CCL2, CCL4, CCL5, CCL7, CCL8, CCL19, CCL20, CCL28, CXCL8, CXCL9, CXCL10*, and *CXCL11* all exhibited an activated expression profile. By contrast *IL16*, a chemoattractant and modulator of T cell activation (29, 30), was downregulated at both 6 and 16 h of infection. Furthermore, for both neonates and adults a downregulation of pro-inflammatory genes (CSF1R, LRRK2, and MAF) (31–33), and genes involved in promoting lymphocyte activation and signaling, such as BLC6, GNAQ, SLA, and SYK was observed (34–40) (Figures 4B and 5B). This suggests that the while a strong IFN response is induced in DCs, an attempt at restricting the immune response is made by suppressing key genes promoting the inflammatory activation of lymphocyte activation and signaling. Together the dual play between inflammatory activation and negative feedback highlight the underlying flexibility in the immunomodulatory response.

The repertoire of IFN antiviral response gene subnetworks, included, but was not limited to, *MX1, MX2, OAS2* and *OAS3, OASL, IFIT1-3, IFIT5*, IF16, IFI35, GBP1 and 2, and *IFITM1-3* (41–47). Notably, USP18 has a key role for potently inhibiting the IFN response maintaining a cellular balance of ISG15-conjugated proteins by cleaving ISG15 fusions (48). Other notable antiviral responsive pathways include TRIM5 and TRIM6 that are also associated with Cell network (see below). TRIM5 and TRIM6, two E3-ubiquitin ligases, have been shown to play a role in retroviral restriction. TRIM5 has been shown to promote premature disassembly of the viral capsid (CA) protein lattice blocking reverse transcription, nuclear entry, and integration (49), delivering the restriction-sensitive retroviral capsids to autophagosomes for destruction (50, 51), and promoting TAK1-dependent innate immune signaling (52) suggesting that TRIM5 acts as an innate immune pattern recognition receptor (PRR) specific for the capsid lattice. Similar to TRIM5, TRIM6 have been shown to display antiviral properties by stimulating IKBKE-mediated antiviral responses including induction of IFN-β (acting downstream of MAVS/IPS-1) and ISGs (including *IRF7* and *ISG15*), but not NF-κB-dependent cytokines (53).

Distinctively, one large cell-cycle network, comprising of clustered gene encoding proteins involved in chromatin remodeling during mitosis and DNA replication and cell-cycle progression, was identified among the upregulated genes (Figure 4A; Table S1 in Supplementary Material). These included, but were not limited to, the genes identified in the pathway analysis (Figures 3B). Moreover, a small network of genes involved in FA metabolism (*ELOVL6, ELOVL7*, and *PTGS2*), was also identified.

Notably several networked (encircled) and disconnected (not shown) G-protein coupled proteins were identified among the downregulated genes (Figure 4B; Table 9), including genes encoding proteins involved in the complement system (*C5AR1*) (54), inflammatory response (*ADORA3, ARRB1*, and *CXCR4*), and *GRK3 (ADRBK2)*, and *RGS18* that function as a beta-adreneric receptor kinase, a receptor for ADP, a receptor for succinate, and as a regulator of G-protein signaling, respectively. Interestingly, ADORA3 has previously been reported to be involved in the regulation of dendritic cell phagocytosis and inflammatory response (55). Glucocorticoid dexamethasone-differentiation of monocyte-derived human DCs was shown to upregulate ADORA3 (and other apoptophagocytic genes) resulting in enhanced capacity of dendritic cells to engulf apoptotic neutrophils and to induce an inflammatory response (55). Moreover, beta-arrestin 1 (ARRB1) has been shown to be involved in IL8-mediated granule release in neutrophils (56) and to regulate IL8-induced CXCR1 internalization in HEK 293 cells (57). The signaling by the chemokine receptor, CXCR4, has been shown to be enhanced by the US27 gene of HCMV product (58). Furthermore, HCMV-encoded UL33 and UL78 have been shown to heteromerize with CXCR4, affecting cell surface expression, internalization, and activity (59).

Overall, the network analysis of up- and downregulated genes in adult DCs revealed, among the upregulated genes, similar networks as those observed in the cord DCs (IFN cluster cIFN6-16h, Cell-cycle network and FA metabolism) consistent with identified over-represented pathways (Figure 3), suggesting a concordance in the cellular response between HCMV-infected cord and adult DCs (Figure 5A). Moreover, similar to cord DCs the same networked and disconnected (not shown) G-protein coupled receptors were also identified in the networked downregulated genes at 16 h of infection (Figure 5B). Interestingly, many of the identified GPCRs have been shown to regulate B and T lymphocyte activation and modulate the Th1/Th2 balance (60–65), suggesting that the downregulation may be associated with limiting viral spread as well as the inflammatory response.

Altogether, these results suggest that HCMV infection in neonatal and adult DCs strongly induce dynamic plastic changes, that affecting cellular gene networks functionally involved in pro-inflammatory antiviral host immune response as well as immune cell expansion.

### Loss-of-Function Screen Identifies Pro- and Antiviral Roles for the Suppression of GPCR Network upon Infection

As described above a notable suppression of GPCRs are found at late times of infection in both neonates and adults. Macrophages and dendritic cells are well known to express a wide range of GPCRs that influence their immune function (66–70). Indeed wide ranges of inflammatory mediators are known to couple to GPCRs, including ATP (71), histamine (72), LPA (73), and sphingosine-1-phosphate (74). By contrast, little is known about anti or proviral functions of GPCRs. Network analysis of the differentially expressed genes in cord DCs, revealed among the downregulated genes after 16 h of HCMV infection several GPCRs with pro-inflammatory response function (*ADORA3, ARRB1, C5AR1, CXCR4, GPR34, GPR183, GRK3*, and *RGS18*) (Figure 4B; Table 9). In addition to these, HCMV infection of cord DCs resulted in a suppression of several other GPCRs [including *GPR68, GPR183 (EBI2)*, and *GPR146*] that, together with *C5AR1, CXCR4*, and *RGS18*, have been shown to be highly enriched in unstimulated macrophages and dendritic cells (67, 69, 70, 75) (Table 9). Among the GPCRs that were found to be upregulated (*CCL2, RGS5, RGS20, GNAI1, GNG11, GPR27, GPR37, GPR161, GPR180*, and *HCAR3*) only *CCL2, RGS5, RGS20*, and *GNG11* have been shown to be expressed in macrophages and dendritic cells and/or implicated in immunity (70, 76–79). *HCAR3* expression has, however, been shown to be induced in breast cancer patient samples, where it plays a role in controlling intracellular lipid/FA metabolism (80). At 6 h of infection, the expression of most, but not all, of the identified GPCRs remained unchanged from mock-infected cells (Figure 4B; Table 9). The expression of *ARRB1* and *GPR146* were both slightly downregulated whereas the expression of GPR34 was downregulated eightfold and the expression of *HCAR3* and *RGS20* upregulated fivefold.

The most highly downregulated GPCR in the HCMV-infected cord DCs were regulator of G protein signaling 18 (*RGS18*) and G-protein coupled receptor 34 (*GPR34*), which have been shown to be significantly expressed in DCs (70, 75). In addition to *RGS18* and *GPR34*, the expression of *ADORA3, ARRB1, C5AR1, CXCR4, GPR68, GPR82, GPR146, GPR183 (EBI2)*, and *GRK3* (*ADRBK2*) was also highly suppressed at 16 h. The same GPCRs identified in cord DCs, with exception for *GNG11* and *HCAR3* (not differentially expressed), were also found to be differentially expressed in adult DCs after viral infection, with some already repressed after 6 h of infection, many unchanged, and *CCL2* and *RGS20* upregulated fivefold to sixfold (Figure 5B; Table 9). As infection progressed in the adult-derived DCs, similar expression changes as those observed in cord DCs were observed for all GPCR genes, but *GNG11* and *HCAR3* that did not exhibit an altered gene expression. Together, these results suggest that while the overall GPCR regulation is seemingly similar between cord and adult DCs, subtle differences, that possibly fine tune the activity of the DC response, exist.

**Figure 6:**
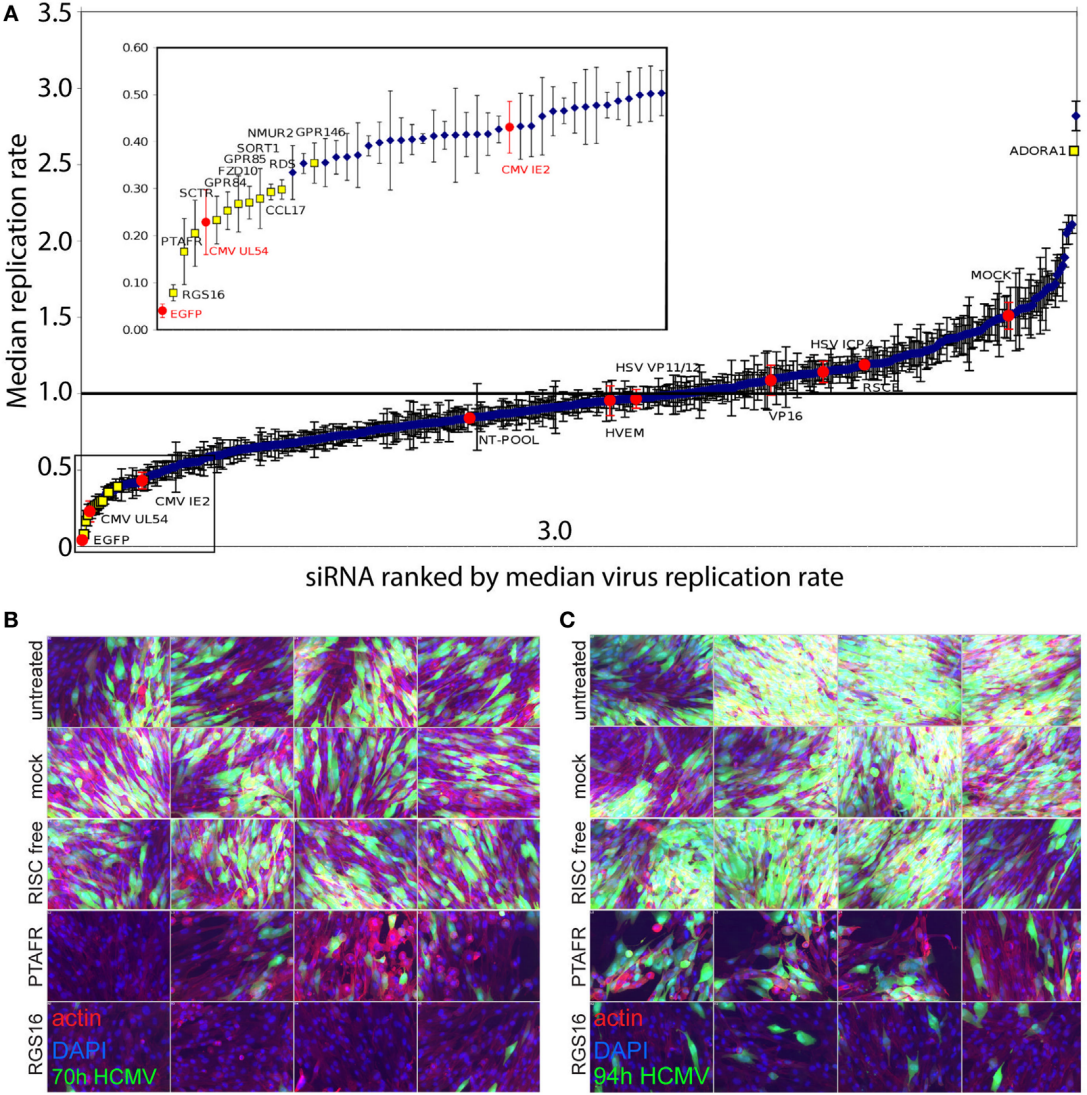
Loss-of-function screen of GPCRs using an HCMV real time replication assay. MRC-5 fibroblasts were reverse-transfected (*n* = 3 with two technical replicates) with siRNA from the Human GPCR SMARTpool library followed by infection with AD169-GFP HCMV. Untreated controls cells, mock treated cells (reagent only), siRNA targeting eGFP, HSV ICP5, HSV VP11/12, HSV VP16, HVEM, Non-Targeting Pool, and cells treated with RISC-free siRNA served as negative controls. Cells treated with siRNA, against IE2 and UL54 served as positive controls. All cells were infected with AD169-GFP HCMV post-siRNA treatment and growth monitored using eGFP detection. Distribution profile of (plate-by-plate negative control-normalized) virus replication slopes across the entire screen [median ± median absolute deviation (MAD)], indicating controls and potential hits of interest **(A)**. High-content images (PerkinElmer OPERA) of siRNA-treated and AD169-GFP-infected fibroblasts at two different time points **(B,C)**. Knockdown of PTAFR or RGS16 was followed by infection with AD169-GFP HCMV for 70 h **(B)** and 94 h **(C)**. Untreated controls cells, mock treated cells (reagent only), and cells treated with RISC-free siRNA, all infected with AD169-GFP HCMV, served as negative controls. Nuclei were stained with 4′,6-diamidino-2-phenylindole (DAPI) and actin was visualized with AlexaFluor-647-Phalloidin.

To further investigate the role of these receptors in HCMV infection, the role of GPCRs on viral replication and spread was functionally investigated. An unbiased Human GPCR SMARTpool RNAi library screen was performed using HCMV-GFP infected MRC-5 human fetal lung fibroblasts (Figure 6). The siRNA library screen consisted of pools of siRNA (4 siRNA duplexes that each target a single cellular gene) targeting a total of 516 individual GPCRs. Screens (*n* = 6) were conducted in three separate independent biological replicate screening assays in order to stringently identify a reproducible high confidence hit. The siRNA-mediated knockdown identified 117 GPCRs that when depleted resulted in a significantly reduced viral replication. Rank-product analysis of the multiple independent GPCR SMARTpool screen statistically identified 11 high confidence GPCR hits (*RGS16, SCTR, PTAFR, GPR84, GPR85, NMUR2, FZD10, RDS, CCL17, SORT1*, and *GPR146*), whose loss of expression resulted in the strongest negative effects on HCMV replication (*p*-value < 0.05) and one hit (*ADORA1*) for antiviral role. While siRNA-mediated knockdown of all 12 hits did not affect cell viability under the conditions tested (Figure 6A; Table 8), further siRNA deconvolution experiments, validating the hits of the library screen identified *CCL17, GPR84, GPR85, PRAFR, RGS16*, and *SORT1* as particularly noteworthy. We find in high content image analysis that siRNA-mediated knockdown results in a visually marked reduced replication and spread of HCMV at late time points of infection (70 and 94 h postinfection) that is independent of cell viability (Figures 6B,C). Out of the 117 candidate genes in the screen, only *CCL2, GPR82*, and *GPR146* were found to be differentially expressed in the HCMV-infected cord and adult DCs.

**Table 8:** Rank-product analysis of GPCR SMARTpool screen results indicating putative hits with the strongest negative effect on HCMV replication (percentage of false positives, pfp < 0.05).

Symbol	pfp value	Median neg-norm virus replication	Median neg-norm cell viability	No. of deconvoluted siRNAs that reduce virus repl. (median + median absolute deviation) by ≥ 45% compared to controls
RGS16	0.001	0.08	1.225	3/4
SCTR	0.008	0.16	0.977	2/4
PTAFR	0.009	0.20	1.422	3/4
GPR84	0.009	0.23	1.038	4/4
GPR85	0.015	0.26	0.958	3/4
NMUR2	0.018	0.27	1.072	2/4
FZD10	0.018	0.24	1.366	2/4
RDS	0.020	0.29	0.680	2/4
CCL17	0.021	0.29	1.086	3/4
SORT1	0.022	0.26	0.907	3/4
GPR146	0.039	0.35	1.345	2/4

**Table 9:** Fold-change expression of GPCR genes in HCMV-infected cord and adult DCs.

Gene	Cord 6 h	Cord 16 h	Adult 6 h	Adult 16 h	In GPCR SMARTpool screen
ADORA3	nd	−9.49	nd	−4.78	
ARRB1	−2.20	−3.49	−2.76	−5.11	
C5AR1	nd	−2.75	nd	−6.14	
CCL2	9.18	6.67	5.62	3.28	*
CXCR4	nd	−7.83	−3.52	−9.93	
GNAI1	nd	12.94	nd	9.38	
GNG11	nd	4.77	nd	nd	
GPR27	nd	4.04	nd	4.14	
GPR34	−7.91	−22.40	−5.69	−13.49	
GPR37	nd	12.41	nd	9.32	
GPR68	nd	−2.04	nd	−3.29	
GPR82	nd	−3.39	nd	−4.63	*
GPR146	−2.02	−3.27	−2.12	−3.29	*
GPR161	nd	3.37	nd	3.98	
GPR180	nd	2.98	nd	2.69	
GPR183	nd	−7.88	nd	−7.60	
GPSM2	nd	4.69	nd	3.74	
GRK3	nd	−6.47	nd	−7.26	
HCAR3	5.60	nd	nd	nd	
LANCL1	nd	4.92	nd	4.24	
P2RY13	nd	nd	nd	nd	
RGS18	nd	−12.58		−12.75	
RGS20	5.62	2.99	5.88	nd	
RGS5	nd	3.21	nd	3.30	
S1PR1	nd	nd	nd	nd	
SUCNR1	nd	nd	nd	nd	

*nd, not detected; >, conversion*.

### Regulatory Pathways for Modulating the IFN Immune Response Are a Central Feature Developed in HCMV-Infected Neonatal and Adult DCs

**Figure 7:**
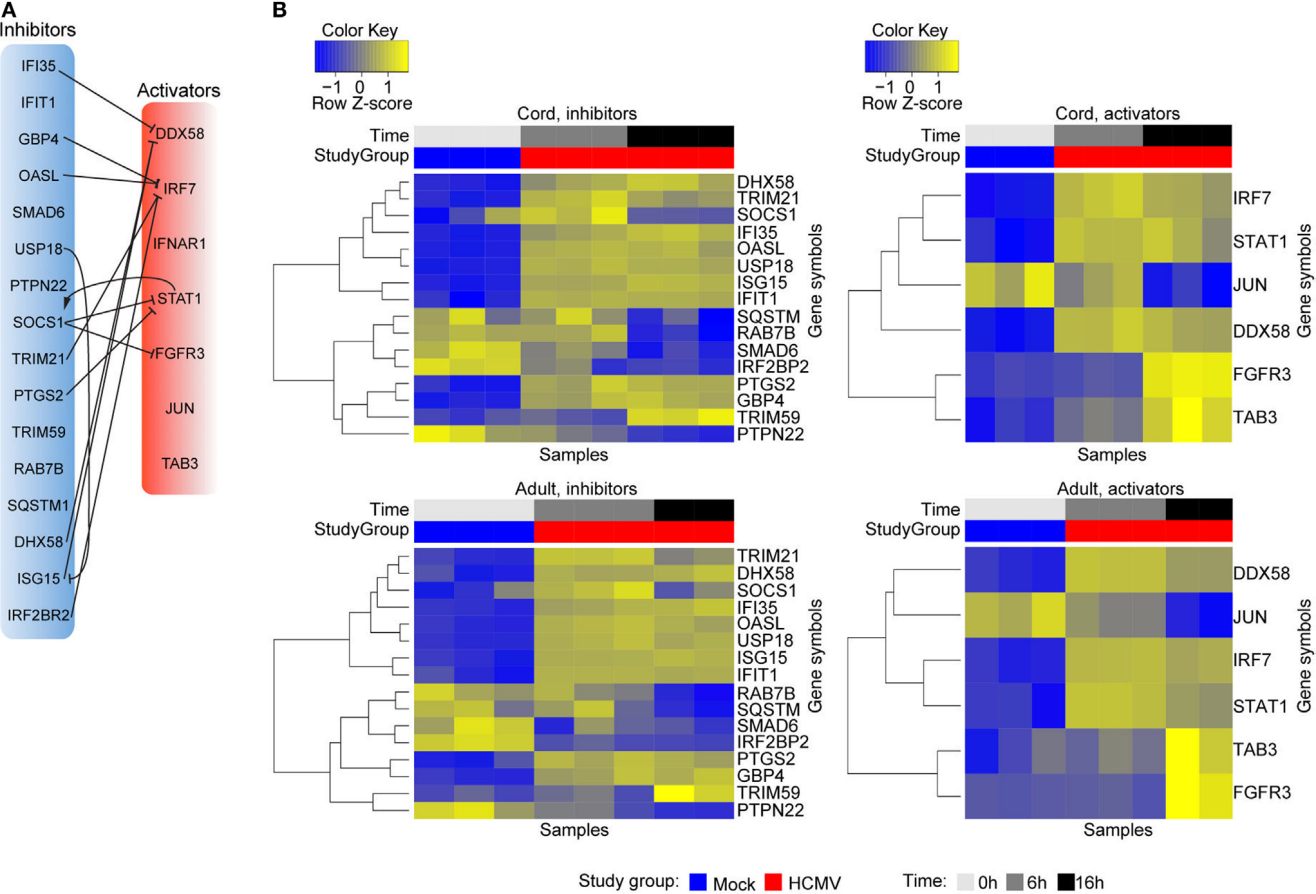
Expression analysis of immune activators and inhibitors reveal a complex regulation of the IFN response, centered on DDX58 and IRF7 activation. The immune inhibitors and immune activators that were identified among the significantly differentially expressed genes, in HCMV-infected cord and adult-derived DCs, and the relationship between them, is depicted in **(A)**. Their per-gene normalized expression in mock-infected (0 h), 6 h, and 16 h postinfection is visualized using hierarchical clustering **(B)**.

A repeating pathway biology theme in the host response to infection is that immune activation pathways are concurrent with immune inhibitory. This counter balance readily establishes feedback networks leading to flexibility and plasticity in response for ensuring host protection but which can also provide an unwanted opportunity to the virus. In this regard the observed activation of genes involved in ISG15 antiviral mechanism and RIG/MDA5-mediated induction of IFNα/β pathways (Figures 3–5; Table S1 in Supplementary Material) results prompted us to further investigate the regulation of these immune response by studying the expression profile of various inhibitors/regulators of the immune response and their targets (Figure 7). For these studies activators and inhibitors of PRRs and IFN receptors and their downstream signaling pathways were investigated. Furthermore, the expression of inhibitors of IFN production, as well as the expression of TRIMs and TRIPs that have been shown to regulate the immune response, was also investigated. Known immune inhibitors such as *SMAD6* that inhibits TGF-beta anti-inflammatory signaling, *PTPN22*, a phosphatase repressor of IFNG activation of myeloid cells, *RAB7B* as a moderator (through lysosome degradation mechanisms) of TLR-IFN responses in human dendritic, and to some extent *SQSTM1* with roles in autophagy and IFNG host defense, were all actively expressed in mock-infected cells, but their expression is repressed upon infection (Figure 7B). *TRIM59* also maintained a repressed state throughout early infection, followed by a strong activation at 16 h of infection. *SOCS1*, encoding Suppressor of cytokine signaling 1, which is induced by STAT1 activation in response to cytokine stimulation, was induced early in infection (6 h), followed by an active downregulation from 6 to 16 h of infection. Furthermore, *PTGS2* involved in the production of anti-inflammatory prostaglandins was also induced upon infection, with possible negative roles upstream and downstream of blocking IFN-STAT1-IRF activation pathways, while *IRF2BP2* a potent repressor of innate inflammation was not (Figure 7B).

In addition, several genes encoding inhibitors of DDX58/RIG-I (*IFI35, DHX58, ISG15*) and IRF7 (*GBP4, OASL* and *TRIM21*) are also upregulated in infected cord DCs (Figures 7A,B) (81–83). Meanwhile, the expression of both *DDX58* and *IRF7*, the master transcription factor for type I IFN, were upregulated at both 6 and 16 h of infection. Activated DDX58 has been shown to interact and dimerize with both MAVS and MDA5 (84), resulting in the activation of TBK1 and IKBKE (IKKE) followed by the subsequent phosphorylation-induced activation of IRF3 and IRF7 and transcriptional activation of antiviral genes including *IFNA* and *IFNB*. IFI35 was recently identified in a genome-wide small siRNA screen as an IFN-induced protein required for vesicular stomatitis virus infection (81). Overexpression of IFI35 was demonstrated to suppress the *IFNB* and *IFIT* promoters. Furthermore, IFI35 was also shown to inhibit activation of DDX58 (inhibit dephosphorylation of DDX58) and to regulate proteasome-mediated degradation of DDX58 (81), overall, implicating IFI35 in negatively regulating type I IFN signaling in a DDX58-dependent manner. DHX58, also known as LGP2, has on the other hand been shown to both positively and negatively regulate DDX58 and MDA5-mediated antiviral signaling (85–87). Similar to *IFI35* and *ISG15*, it showed a consistent infection-induced transcriptional response. Its inhibitory action on DDX58 signaling may involve competition with DDX58 for binding to (1) the viral RNA (88), (2) binding to DDX58 and, thus, preventing dimerization between DDX58 and MAVS (82), or (3) competing with IKBKE (IKKE) for its binding to MAVS, subsequently inhibiting activation of IRF3 (89). Finally, *ISG15* encodes an ubiquitin-like protein that has been shown to exhibit antiviral activity toward several viruses including Influenza A, HIV-1, and Ebola virus (90–92). It conjugates (ISGylation) to intracellular target proteins upon activation by IFNα and IFNβ (83, 91, 93–98). In addition to DDX58, ISG15 has been shown to target MX1 and EIF2AK2 (83), which were modified (upregulated) in the infected DCs. Notably, ISG15, is a target of USP18, a type I IFN-induced ubiquitin specific protease that effectively cleaves specifically ISG15 fusions (Figure 7). In the HCMV-infected cord and DCs, *USP18* was also upregulated. *USP18*-deficient mice have been shown to demonstrate a large increase in ISG15-conjugates (99) as well as a hypersensitivity to IFN, linking USP18, as well as ISG15, to negative regulation of the type I IFN response (100). *GBP4, OASL*, and *TRIM21*, which have been shown to posttranslationally modify the activation and function of IRF7 (101–104), all displayed an infection-induced expression (Figure 7B).

Guanylate-binding protein 4, GBP4, has been identified as a negative regulator of the virus-induced type I IFN response, by disrupting TRAF6-mediated IRF7 ubiquitination (101). Of note, the expression of both *TRAF3* and *TRAF6* were unaffected by the infection (expressed below the threshold of detection). *TRIM21*, which was upregulated by the infection, encodes an E3-ubiquitin ligase that has been shown to, upon interaction with FADD, ubiquitinate IRF7 (103). Ubiquitination of IRF7 was shown to affect the phosphorylation status of IRF7 and its interaction with TRAF6, resulting in a repressed IFNα expression in Sendai-infected cells (103). TRIM21 has, furthermore, been shown to regulate the type I IFN signaling pathways in human microglial cells infected with Japanese encephalitic virus, which induces an innate immune response by increasing the production of IFNβ *via* IRF3 activation and phosphorylation (105). Finally, human 2′-5′-Oligoandenylate Synthase Like (OASL), on the other hand, has been shown to exhibit antiviral activity by enhancing specifically DDX58 signaling by mimicking polyubiquitin (104), distinguishing it from the murine OASL1 protein that negatively regulates type I IFN upon viral infection by inhibiting the translation of IRF7 mRNA (102). Loss of *OASL* expression has been shown to reduce DDX58 signaling and enhanced viral replication.

In addition to genes encoding negative immune modulators several genes encoding immune activators [fibroblast growth factor receptor 3 (*FGFR3*), *JUN, STAT1*, and *TAB 3*] and co-stimulators/inhibitors (*DDX58* and *IRF7*) were likewise differentially expressed in the infected cord and adult DCs. In addition to *DDX58* and *IRF7*, the expression of *STAT1*, regulated through positive feedback by IRF7 (106), was also activated and maintained throughout the infection. Notably, *JUN* was activated in mock-infected cells and maintained throughout early infection (6 h), but repressed as the infection progressed (16 h). Concurrently, *FGFR3* and *TAB 3* maintained a repressed state until late infection (16 h) (Figure 7B). While a direct immunological role for FGFR3 has not yet been described, this tyrosine-protein kinase has been shown to play an essential role in the regulation of cell proliferation differentiation, and apoptosis. Moreover, SOCS1 and SOCS3 (not detected) have been shown to interact and modulate FGFR3 signaling in chondrocytes (107), blocking MAPK cascade signaling. Furthermore, FGFR3 activation of the MAPK cascade, albeit in the context of multiple myeloma, has been shown to block the promoter function, expression, and secretion of the chemokine CCL3 (108), linking both SOCS1 and FGFR3 to the modulation of the chemokine response.

The concurrent regulation of activators and inhibitors of the DCs likely represent both host and viral driven responses and are especially reflective for a tunable level of flexibility in developing a plastic response to HCMV infection that can be robustly regulated upwards or downwards.

### Strong Concordant Pathway Biology Responses to Infection Predominate over a Minimal Subtle Difference in Programming between Neonatal and Adult DCs

**Figure 8:**
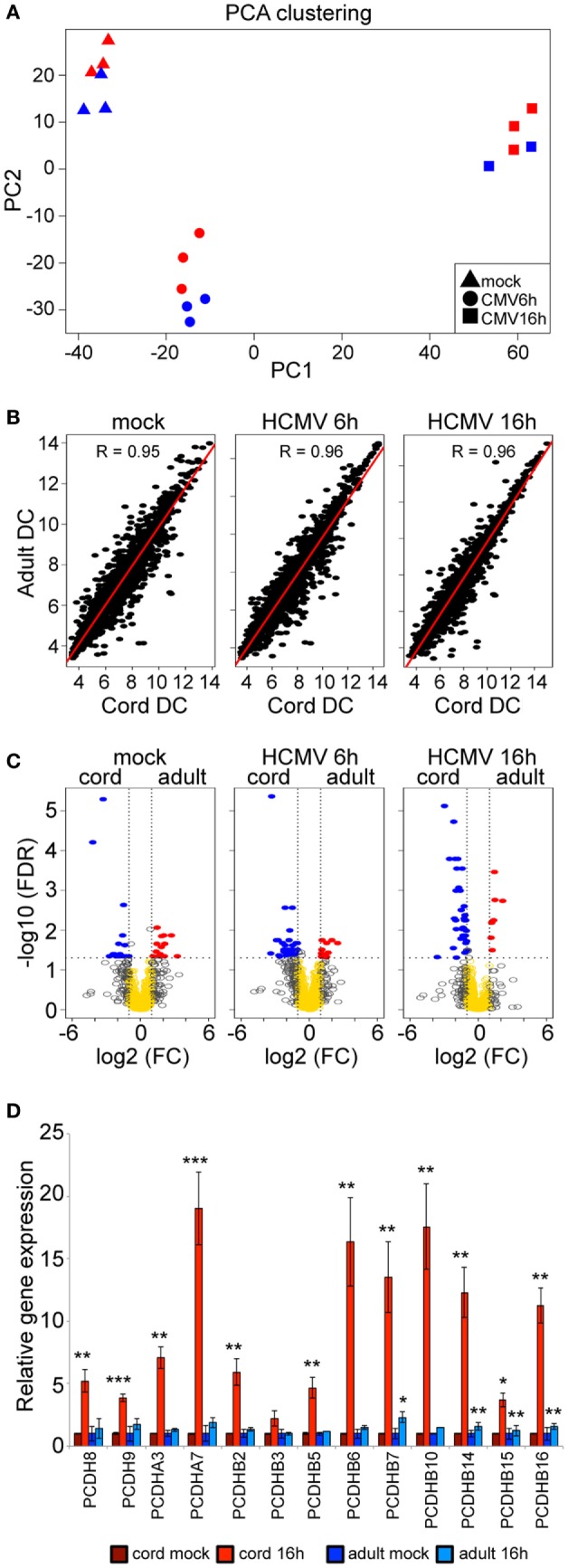
Cord and adult DCs show a high concordant genome-wide response to HCMV infection but differential expression of clustered and non-clustered *PCDH* genes late upon HCMV. PCA clustering over time of mean whole-genome gene expression (log_2_ mean expression) in mock-infected and HCMV-infected neonatal cord (red) and adult (blue) DCs **(A)**. Regression analysis of mean whole-genome gene expression (log_2_ mean expression), between cord and adult DCs, show a strong correlation in mock (*R* = 0.95) and at 6 h (*R* = 0.96) and 16 h (*R* = 0.96) of infection **(B)**. Volcano plots comparing log_2_ mean expression values of filtered genes between adults and cord DCs, after false discovery correction (−log_10_ FDR) **(C)**. Fold-change expression levels of *PCDH* genes in cord (red bars) and adult DCs (blue bars) 16 h of HCMV infection, relative to mock-infected cord and adult DCs (set as 1) **(D)**. The error bars indicate the SEM. Statistical significance was calculated using non-paired student *t*-test. *p < 0.05, **p < 0.01, and ***p < 0.001.

The above analyses show, rather than the anticipated strong hyporesponsive mode of neonatal DCs to infection, a strong and robust activation of host protective and immunomodulatory functions. This raises the question of how similar or different the overall quantitative and qualitative responses are between cord and adult-derived DCs. To quantitatively investigate the similarities and dissimilarities in the response between cord and adult DCs, we first applied a principal components analysis for all samples tested. Figure 8A clearly shows that samples closely grouped with regard to time of infection and not by cord or adult origin. This outcome is consistent with the pathway investigations described above in HCMV-infected cord and adult DCs suggestive of concordant pathways alterations, involving a similar number of genes within each over-represented pathway. Most strikingly and not fully anticipated we further find an extremely high correlation exceeding *R* = 0.95 between the programme of gene expression between neonates and adults cells at each point of infection (mock, 6 and 16 h postinfection, respectively) (Figure 8B). Hence, we conclude that there exists a high similarity in the immune response against HCMV infection in both neonates and adults.

Notably, the high correlation between neonatal and adult mock-uninfected DCs points to an almost identical molecular phenotype. Accordingly, we next proceeded to test directly for any statistically significant differentially expressed genes using an empirical bayes test adjusted for false discovery. We find only 33 genes with an adjusted *p* < 0.05 and twofold change in gene expression between mock-uninfected adult and cord DCs (Figure 8C, mock panel, Table S4 in Supplementary Material). These genes do not form any defined network or pathway; however, at the gene analytic level there are few differentially expressed genes worth commenting. Specifically, the basal expression levels of *RSAD2*, one of the most strongly type I IFN inducible genes, is significantly higher in adult DCs. This might suggest a resting level that could be reflective in adult DCs for a possible primed or increased tonic state in comparison with cord DCs, although this represents the only IFN inducible gene identified. There are also two anti-inflammatory associated genes, *PLA2G5* and *DDIT4L*, which are more highly expressed in the neonatal DCs. These data suggest that if there is an elevated anti-inflammatory state, in cord DC in comparison with adult-derived cells, it is extremely subtle and very weak but plausibly could have an impact upon the speed of immune activation. Consistent with this notion, the pathway biology analysis identified a potent IFN response with altered kinetics in adults and cord-derived cells for a broad expression pattern of type IFN genes (Figures 3–5; Table 1). Similar to cord DCs that expressed a modest number of type I IFN genes (*IFNA1* and *IFNB1*) at 6 h, the expression of IFN genes in adult DCs was also restricted to type I IFN genes (*IFNA1, IFNA13*, and *IFNB1*). At 16 h of infection, the IFN repertoire in adult DCs increased both in numbers of genes and in expression strength (*IFNA1, IFNA2, IFNA4, IFNA5, IFNA7, IFNA8, IFNA10, IFNA13, IFNA14, IFNA16, IFNA21, IFNB1, IFNG*, and *IFNW1*), consistent with the observations in cord DCs. While gene induction in adult DCs was higher than in cord DCs, broadly the same genes are induced in both cell types late in infection, with exception that in adult DCs a statistically significant increase in *IFNG* is seen. Notably, for both cord and adult DCs, expression of *IFNB1* was initially induced at 6 h followed by a marked repression, while *IFNA1* was the strongest induced gene in both adults and cord DCs at late times.

At 6 and 16 h postinfection a comparison between infected cord and adult-derived populations showed a slight increase in statistically significant differentially expressed genes accounting for 46 and 43 genes, respectively (Figure 8C). Again these genes lists (Table S4 in Supplementary Material) do not represent any selective pathway or network; however, at the gene analytic level there is a far greater percentage of significant genes activated in the cord cells. At 6 h postinfection a number of markers that have the capacity to contribute an anti-inflammatory role are expressed at a slightly higher level in the neonatal cord cells in comparison with adults, and include *CD200, ALOX5*, and *CHI3L1*. These markers may highlight a very subtle elevation in negative feedback with potential impact in altering the reference level of the set-point for triggering the adaptive immune arm in neonatal cord cells. At 16 h the multifaceted Chitinase-3-like-1 protein (CHI3L1) marker remains elevated in neonatal cells. However and most strikingly, 12 members of the multigene protocadherin (PCDH) family were highly upregulated 16 h post HCMV infection in cord DCs (Figure 8D). PCDHs comprise a large group of transmembrane proteins within the cadherin superfamily. Cadherins are calcium-dependent adhesion glycoproteins involved in the regulation of cell–cell contacts (109). The PCDHs include >80 molecules, expressed mainly in the vertebrate nervous system, that play several important roles in the development of the brain, in the organization and formation of neural circuits, and in the regulation of brain function and dendritic development (110–112). Also, it is reported that some PCDHs may act as tumor suppressors, inhibiting the proliferation and metastasis of cancer cells (113, 114). Both *PCDH8* and *PCDH9*, two non-clustered PCDHs, were found to be upregulated in the HCMV-infected DCs. PCDH8 has been shown to be involved in synaptic plasticity of dendritic cells and represents a linkage to schizophrenia (115, 116). The PCDH9 is also related with a neurological disease and autism spectrum disorder (117). Both PCDH8 and PCDH9 have, furthermore, been shown to be mutated or downregulated in a variety of tumors (118–120).

In addition to the non-clustered *PCDH8* and *PCDH9* genes, several clustered PCDH genes were upregulated after 16 h of infection (Figure 8D). To date most PCDH studies are related to neurological functions (121–125). Remarkably, the combinatorial expression patterns of the clustered PCDHs can generate enormous cell surface diversity, with 3 × 10^10^ variations for each individual neuron. This type of diversity on DCs may impart immune modulatory interactions. Taken together, this suggests that the PCDH genes, upregulated in HCMV-infected cord DCs, could play an important role in yet to be defined immune cell–cell interactions and possibly the clinical neurological diseases that can appear during the congenital infection.

Overall we conclude that neonatal derived cells are relatively unrestricted and imbued with a full plastic capacity to be programmed in a similar manner to adult-derived cells upon infection. However, this response is not completely identical and a very small number of gene-specific significant differences are apparent. Further, while statistically not significant, after relaxing false discovery correction, type I IFN gene networks are found to be temporally and quantitatively differentially expressed between cord and adult cells. This highlights a rather subtle difference in negative feedback that may have a role in governing the reference level of the set-point for DCs effector functions. Nevertheless, these results clearly highlight that neonatal DCs have the full capacity to mount robust effector host pathway response as adult DCs.

## Discussion

Here, we highlight that HCMV is an excellent marker of human immune health developing benign infections for those with a healthy immune system but presents with devastating consequences for an immune compromised patient. The developing fetus and neonates are an especially vulnerable population with HCMV remaining a leading cause of congenital infection. The overall birth prevalence of congenital HCMV infection is approximately 0.5% but with the majority (90%) of these infections developing no major sequelae. Accordingly, this points to an underlying resilience in immune health for this highly susceptible population that is poorly understood and which is inconsistent with existing dogma about the immune mechanisms governing early-life immunity.

We find through a case investigation of the systemic response of a congenitally infected preterm infant a highly robust myeloid and non-conventional T cell response showing anticipated strong type I IFN antiviral response. Of the myeloid cells the dendritic cells show a significant change. While our present investigations are limited to a single case, further infected cases will need to be studied before any general conclusions can be drawn. Nevertheless, it is well known that DC play a critical role in sensing and driving immune responses to viral infections. Indeed, there is good evidence linking DC Type I IFN response to regulate the adaptive T and B cell with the accepted view that neonatal DCs and other innate immune cells respond sub-optimally to infection *via* differential TLR stimulation (126–128). Many of these studies use gene or protein analytic investigations and only limited work applying a more global system level investigation [e.g., (129)] has been presented. Nevertheless, to date the consensus view is that neonatal cells have a propensity to develop a hypo-inflammatory state, that impact on lymphoid cells as well as the possible involvement of myeloid-derived suppressor cells (130–132).

In our investigation of neonatal cord and adult DCs for genome-wide molecular pathway biology responses we strikingly find a very similar overall vigorous response between neonatal and adult-derived DCs, with only minor and selective temporal differences revealed in gene expression changes upon HCMV infection. While these findings warrant further investigations to increase statistical power to draw general conclusions, a very recent study (10) examining fetal and adult DC populations (through flow cytometry and transcriptomics) found *R* > 0.92 correlation of gene expression profiles comparing different adult and fetal DC populations. Furthermore, functional activation assays showed comparable behavior between fetal and adult DCs, suggesting that fetal DCs are capable of mediating a strong innate immune response. However, in those studies fetal DCs but not adult DCs were found to express higher levels of ARG2 that had a regulatory impact on T cell activation; a finding consistent with the set-point hypothesis described in more detail below. In the case of the neonatal and adult DCs infected with HCMV, both significantly induced ARG2 to comparable levels. Notwithstanding the similarity in ARG2 expression we do detect very subtle and specific kinetic differences in IFN-responsive genes in HCMV-infected DCs between neonates and adults and which may differentially impact immune stimulatory and suppressive responses. We conclude from our, *in vivo* and *in vitro*, investigations of early-life infection with HCMV leads to a strong and flexible innate immune response and which, on the basis of existing dogma for neonatal immunity, is unexpected.

### Signal Immune-Metabolic Innate Pathway Biology Responses to HCMV Infection in Dendritic Cells

The central hallmark signature observed for both adult and neonatal cord DCs is type I IFN responses. The IFN responses represent an important protective antiviral response in dendritic cells. It is notable that while the magnitude of the IFN response is similar, inclusive of TLR gene expression; however, a kinetic difference is observable with a more immediate response detectable in adult DCs that might be suggestive of a more exquisite TLR stimulation capacity in adults in comparison to neonates. Indeed, small quantitative but specific differences in the expression of myeloid cells are known to impact on co-stimulatory and immune-suppressive functions (133). However, it is also noteworthy that the overall scale, magnitude and pathway biology are strikingly similar between cord and adult DCs and is a strong mark of a plastic response.

While there is a robust pro-inflammatory innate immune activation equally observed with infected cord and adult DCs, other major pathways of note are associated with cell cycle, GPCR signaling and metabolic pathway alterations. Of the metabolic pathways major alterations are seen for pathways centered on lipid metabolism, in particular upregulation of sterol (in particular oxysterol metabolism) and long-chain FA synthesis. Notably these pathways are more prominent in adult DCs and are suggestive of known proviral or immune-metabolic responses. In contrast a key unknown network downregulated upon infection is a GPCR network. To explore the functional significance of the GPCR network to HCMV infection we conducted an unbiased systematic viral infection screen of siRNA knock down of 516 known GPCRs. Notably, while a few displayed antiviral roles (e.g., ADORA1) a far greater number were found to contribute to a proviral role, in particular GPR146, RGS16, and PTAFR. These targets identify new previously unknown host-dependent genes/pathways that provide excellent drug targets for inhibiting viral replication.

### Myeloid Dendritic Cell (DC) Inflammatory Responses That Distinguish the Set-Point Hypothesis Mechanism from Immune Immaturity in Neonates

The susceptibility to infection in early life is often assumed to be due to developmental *immaturity* of the immune system and as a consequence is hyporesponsive to infection leading to an overall insufficiency toward protection. As discussed this view has been primarily generated through selective analysis of a number of inflammatory mediators and there has been limited systematic investigation through unbiased global analyses. Critically, the immaturity mechanism fails to fully account for resilience. As previously mentioned, there is increasing evidence pointing to an alternative mechanistic understanding that we have previously proposed to involve a set-point control system in the regulation of the immune response to infection. The set-point hypothesis readily accounts for both susceptibility and resilience through altering the reference level for triggering the adaptive immune response (11). In this regard, innate immune myeloid cells, in particular dendritic cells, play a critical role as early sensors and effectors of the immune response to generating the set-point reference level in viral infection.

**Figure 9:**
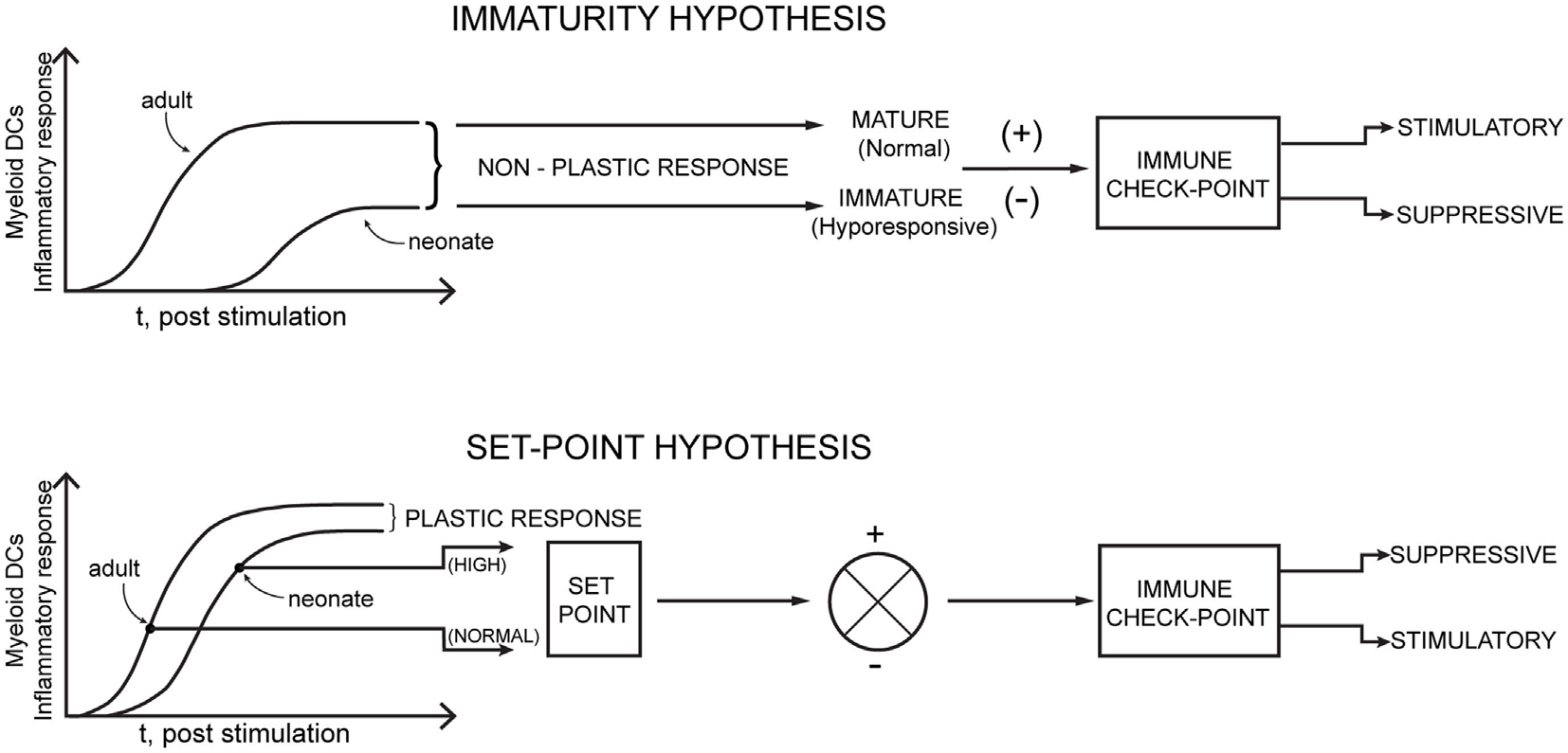
Summary figure.

Figure 9 shows a schematic summary of the temporal DC response to infection and the differential outputs in the context of the *immaturity hypothesis* and the *set-point hypothesis*, in neonates and adults. In the scenario of “immature” DC, the programmatic inflammatory response to infection is inherently lower (hypo-inflammatory) both in terms of kinetics of response and in magnitude for neonatal cells that consequently fail to trigger the adaptive arm; while adult DCs produce a robust normal response for triggering T and B cell effectors, *via* immune check-point control interactions. By contrast, in the case of the *set-point control* mechanism, the programmatic inflammatory response to infection in neonatal cells is expected to be similar in magnitude to adults but with slightly altered kinetics. This is likely generated through subtle and variable negative feedback, leading to a set-point reference with a variable range from normal to high for triggering T and B cell responses.

The distinguishing hallmark feature between these two mechanisms is that the genomic programming (measurable at the transcriptomic level) is in the case of the immaturity hypothesis non-plastic while for the set-point hypothesis develops a highly plastic response that would be anticipated to be functionally protective and thereby aiding resilience. However, a high set-point reference maintained through increased negative feedback may also further promote an inhibitory immune check-point control of the adaptive arm leading to an immunosuppressive state with an increased susceptibility to infection and disease. In this study, we find clear evidence for a plastic response in infected neonatal DCs consistent with the set-point hypothesis model rather than immaturity model. Further, we also note that the set-point hypothesis is also consistent with a recent seminal study revealing in dendritic cells homeostatic immune-suppressive responses in fetal life during gestation but not for adult cells (10).

### Concluding Remarks

In comparison with adults the natural history of neonatal HCMV infection is clearly associated with higher risk of susceptibility to disease yet there is a previously neglected consideration of resilience that, epidemiologically reveals little or no clinical illness. On balance this neglected aspect of resilience to HCMV infection in early life indicates that neonatal immune health has, contrary to the current dogma of immune deficiency, the capacity to mount a plastic protective immune response to infection. Here, we report, through an unbiased investigation, molecular evidence based on computational pathway biology of neonatal HCMV infection that supports rather than refutes this proposition. Moreover, these findings together with recent published work add further weight toward supporting regulatory mechanisms consistent with an altered homeostatic set-point in myeloid immune stimulatory and suppressive functions rather than developmental immaturity. Critically, unlike the immaturity hypothesis, the set-point hypothesis can account for both susceptibility and resilience to infection.

Specifically we find in a systemic congenital infection response a significantly marked activation of myeloid dendritic cells as well as markers for NK and/or non-conventional T cells. These HCMV associated molecular-cellular systemic responses are in marked contrast to neonatal bacterial infections that show little activation of dendritic cells but which instead exhibit marked changes in monocytes and neutrophils. Moreover we unexpectedly find that the cell intrinsic programming ability of neonatal and adult-derived DCs are similar; revealing only very subtle changes associated with the regulatory and temporal feedback control of immune activation. Overall, the *in vivo* and *in vitro* findings highlight that neonatal immunity is relatively intact demonstrating plasticity to mount effective host responses but which may result in subtle regulatory mechanistic differences in temporal and feedback control.

In summary, our findings are consistent with the previously proposed hypothesis that neonatal immunity is not overly impaired but may have a different homeostatic set-point in controlling key immune checkpoint stimulatory and inhibitory pathways. We believe HCMV neonatal infection provides a valuable clinical disease model to examine set-point control in neonates and to illuminate central control mechanisms of neonatal immunity through studying asymptomatic and symptomatic neonates.

## Materials and Methods

### Human Cord Monocyte-Derived Dendritic Cell (DC) and Human Whole Blood Gene Expression

Data for the cord and adult monocyte-derived dendritic cells (DCs) gene expression analysis were previously published by Renneson et al. (14), and can be accessed at the Expression Omnibus (134), hosted by the National Centre for Biotechnology Information, under accession number GSE101855. All data for the human whole blood gene expression analysis can be accessed at the Gene Expression Omnibus (134) under accession number GSE25504.

### Statistical Analysis

Data processing and analysis for transcriptomic data were performed with R Language and Environment for Statistical Computing (135).

For quality control, and data processing, Bioconductor packages (136) was used. Quality control and data processing determined one adult sample (GSM2717282) as an outlier, and for this reason it was excluded from further study, giving a final sample number for mock-infected cord (*n* = 3) and adult (*n* = 3) DCs, 6 h HCMV-infected cord (*n* = 3) and adult (*n* = 3) DCs, and 16 h HCMV-infected cord (*n* = 3) and adult (*n* = 2) DCs. The arrayQualityMetrics package (137) was used to determine data quality. Background correction and normalization was done using the RMA (138) algorithm. For further downstream analysis in R, first, a low constant (value = 1) was added to all normalized log_2_ expression values to eliminate negative values generated as an artifact during background correction. Next, a non-specific filter was applied to remove gene probes with low variance across all samples in the study (SD ≤ 0.4) and to further remove gene probes without existing transcript annotation, both in order to reduce the number of non-informative gene probes affecting multiple-testing adjustment subsequent to statistical testing. To remove redundant gene probes (per gene), each gene was represented a gene probe with the maximum coefficient of variation (CV). The per-gene hypothesis of differential average expression was tested between the HCMV-infected groups and the control group, using the empirical Bayes moderated t-test (limma package) (139). Resulting *p*-values were adjusted for multiple testing using the Bonferroni–Holm algorithm (140). Genes with an absolute log_2_FC ≥ 1 and an adjusted *p* < 0.05 were considered statistically significant.

Analyses of the human neonatal study, including 1 HCMV-infected neonatal patient sample (Inf-075, GSM627045) and 13 Coagulase negative *Staphylococcus* samples [Inf084 (GSM627048), Inf-089 (GSM627049), Inf114 (GSM627054), Inf116 (GSM627055), Inf119 (GSM627056), Inf125 (GSM627057), Inf133 (GSM627059), Inf138 (GSM627060), Inf152 (GSM627063), Inf157 (GSM627064), Inf162 (GSM627066), Inf198 (GSM627069), and Inf203 (GSM627070)], were done as described (9). All downstream analysis of the human neonatal data was performed on per-sample *z*-score transformed values (mean = 0, SD = 1) for direct comparability.

Cell type enrichment analysis, visualized with distribution plots, was based on manually procured lists of cell type specific marker genes (Table S2 in Supplementary Material) and was performed on significantly differentially expressed probes that were manually procured from the *z*-score transformed neonatal array [Sample Inf-075 and all control samples (GSM627008–GSM627042)]. The initial numbers of cell markers, for each cell type, were for B cells (381 genes), T cells (244 genes), dendritic cells (72 genes), monocytes (230 genes), neutrophils (837 genes), and NK cells (77 genes). The number of genes, for each cell type, after (a) mapping of marker lists to the neonatal array platform and (b) exclusion of invariate markers, resulted in a reduced number of cell markers that were subsequently used for generating the distribution plots. The final number of genes for each cell type was for B cells (144 genes), T cells (225 genes), dendritic cells (44 genes), monocytes (211 genes), neutrophils (750 genes), and NK/NCT cells (59 genes). Shapiro–Wilks test was used to establish if these data follow a normal distribution, informing the decision to compare collective markers for a given cell type were then compared between control and HCMV using non-parametric Wilcoxon Rank Sum tests. FDR correction using Bonferroni was done on *p*-values produced using Wilcoxon Rank Sum tests. Cell marker expression analysis in controls (average *z*-score of 35 controls), Inf-075, and the 13 coagulase negative *Staphylococcus* samples, visualized with technical dot plots, was performed on the same manually procured from the *z*-score transformed neonatal arrays (see Table S2 in Supplementary Material for lists of cell markers), and the median was calculated.

Comparative analysis of the expression of manually procured genes for immune inhibitors (*n* = 15 genes) and activators (*n* = 7 genes) (141, 142), investigated using per-gene normalized hierarchical clustering, was done on the HCMV-infected cord DCs (6 and 16 h) and mock-infected DCs controls.

For comparative analysis of genome-wide response to HCMV infection in mock-infected and HCMV-infected cord and adult DCs, cord and adult samples were normalized and filtered together. A filter of CV > 0.1 (CV of a gene across all samples was required to be > 10%) was applied. Regression analysis was applied to assess the correlation of fold-change values between cold and adult samples.

### Network and Pathway Analysis

Computational network-based approaches were used to examine the relationships in the data using correlation of gene expression and biological relationships. InnateDB^1^ (143–145) and STRING^2^ (146) were used to examine biological network relationships and association with known pathways [REACTOME (20, 21)] among differentially expressed genes (absolute log_2_FC ≥ 1, adjusted *p* < 0.05) in infected cord DCs at 6 and 16 h of infection. STRING analysis of the 114 top differentially expressed genes (absolute log_2_FC > 1.58, adjusted *p* < 0.05), upon congenital neonatal infection, was performed on *z*-score transformed values from patient arrays GSM627045 (114 genes). To examine biological network relationships in STRING, “experiments,” “co-expression,” and “databases” were chosen as active interaction sources, and a minimum required interaction score of 0.400 (medium confidence) was used for all analysis. Identified members within subnetworks, and their fold-change expression, are listed in Table S1 in Supplementary Material. Pathway analyses were carried out step-wise using a pathway biology approach, becoming more focused. Back-transformed fold changes for differentially expressed immune effector genes and metabolic genes (absolute log_2_FC ≥ 1, adjusted *p* < 0.05) are presented in Tables 1–9.

### MRC-5 Human Fibroblasts

MRC-5 human lung fibroblasts (ATCC^®^ ATCC-CCL-171™) were maintained in EMEM (Lonza AG, Basel, Switzerland) supplemented with 10% Fetal Calf Serum (Lonza AG), 2 mM l-glutamine (Lonza AG), MEM Non-Essential Amino Acids (Lonza AG), and 50 U/ml penicillin/streptomycin (Lonza AG). Cells were transferred to DMEM (Lonza AG) supplemented with 10% Fetal calf serum (Lonza AG), 2 mM l-glutamine (Lonza AG), and MEM non-essential amino acids (Lonza AG) (henceforth referred to as complete DMEM media), at least 24 h prior to reverse transfection, and used by passage 33.

### siRNA

The Human GPCR SMARTpool library (GE Dharmacon, Lafayette, CO, USA; Cat#G-003600; Lot#050915) consists of mixtures (pools) of four siRNA duplexes each targeting a single cellular gene (see Table S3 in Supplementary Material for list of genes targeted). To validate the results of the original SMARTpool screen, the following deconvoluted SMARTpools (i.e., sets of four individual siRNA duplexes corresponding to the original SMARTpools) were ordered for following targets (GE Dharmacon): ADORA1 (MU-005415-03), CCL17 (MU-007828-01), FZD10 (MU-005500-01), GPR146 (MU-005549-01), GPR84 (MU-005604-00), GPR85 (MU-005605-01), NMUR2 (MU-005671-00), PTAFR (MU-005709-00), RDS (MU-011102-01), RGS16 (MU-008386-01), SCTR (MU-005724-03), SORT1 (MU-010620-01). Deconvoluted SMARTpools were first screened as individual duplexes (3 biological replicates with 2 technical replicates/biological replicate) to validate the results of the original SMARTpool screen, and then sets of four duplexes were combined in equal proportions to form “reconstituted SMARTpools” for further validation experiments. siRNA controls used were as follows (GE Dharmacon; catalog numbers or custom sequence as indicated): eGFP (P-002102-01-20), IE2 (ggaagaacagggugaagaauu), UL54 (cgauagaacaugagaggcauu), HVEM (M-008096-00), Non-Targeting siRNA Pool #1 (D-001206-13-20), RISC-Free (D-001220-01-20), ICP4 (gcggcgacgacgacgauaa), VP16 (gggcgaaguuggacucgua), VP11/12 (ggacucagccggugacaua).

### Reverse Transfection and HCMV Infection of MRC-5 Fibroblasts

MRC-5 cells were seeded in Corning^®^ 384-Well Optical Imaging Flat Clear Bottom Black Polystyrene TC-Treated Microplates (Corning Inc., Corning, NY, USA) in complete DMEM media and reverse-transfected, in accordance to the manufacturer’s instructions, using 0.2% DharmaFECT-1 (GE Dharmacon) and 25 nM siRNA (individual duplexes from deconvoluted SMARTpools, GE Dharmacon) in a 60 μl final well volume (concentrations indicated are final concentrations). For The Human GPCR SMARTpool library screen of siRNA-transfected cells, three completely independent screens were prepared using technical duplicates, giving a final number of *n* = 6. To determine effects by the individual siRNAs and cell viability in parallel, half of the plate was used for viral infection and the other half, left uninfected, was used to determine cell viability.

For deconvoluted siRNA screens, three identical replicate plates were prepared with siRNA-transfected cells, from which one was used for cell viability (uninfected), and two were subsequently infected to determine the effects on viral replication. In short, 3 × 10^3^ cells were seeded in a volume of 40 μl/well in 384-well plates containing 10 μl DharmaFECT-1 diluted in Hanks Balanced Salt Solution (HSBB) (Lonza AG) and 10 μl siRNA diluted in 1× siRNA buffer (GE Dharmacon). Transfected cells were allowed to recover for 48 h before infection with HCMV. Following reverse transfection, MRC-5 fibroblasts were infected with AD169-GFP HCMV virus (147) at an MOI (0.5), diluted in phenol-red-free complete DMEM media.

### Analysis of Viral Replication and Cell Viability Assay

For The Human GPCR SMARTpool library screen three plates were simultaneously prepared and half of each plate was infected. Virus and cell viability measurements for each plate were simultaneously measured once as an endpoint at 40, 70, or 94 h postinfection. For deconvoluted siRNA screens, cells viability was measured in every screen repeat using a dedicated replica plate at 48 h post-transfection, while viral replication of the remaining two plates were measured repeated for GFP over time-course, to calculate slope values of GFP expression. Linear regression was applied to determine the viral replication (slope) between 30–75 h postinfection. For both The Human GPCR SMARTpool library screen and the screen with the deconvoluted siRNAs, cell viability was measured in using a dilution of 5 μl CellTiter Blue (G8080, Promega, Madison, WI, USA) in 60 μl phenol red-free DMEM (Lonza AG), supplemented as described above, in a 384-well plate. Cell viability was assayed by measuring levels of fluorescent resorufin produced by metabolically active cells using a BMG LABTECH POLARstar Optima plate reader (fluorescence bottom-read; excitation filter A-560; emission filter 590; gain set to 1,500). For siRNA screens, cell viability was measured in every screen repeat using a dedicated replica plate at 48 h post-transfection (the remaining two replica plates in each screen repeat were then infected and assayed for virus replication rate as described above).

### Analysis of the Human GPCR SMARTpool RNAi Screen Data

Subsequent to averaging technical replicate runs for each biologically independent screen, analysis-ready data consist of measured viral growth slopes for each gene in three independent biological replicate experiments. Using R Language and Environment for Statistical Computing (135), we aimed to identify suppressed genes that result in a consistently high or low ranking (with respect to other genes in a given biologically independent experiment run) of their viral growth slope across all three biologically independent runs. We use a Rank Product (148) one-class test statistic to obtain a significance estimate for each gene, including a multiple testing (false discovery rate) adjustment of the *p*-value.

### High-Content Imaging of HCMV-Infected Fibroblasts

Reverse-transfected and HCMV-infected MRC-5 cells were imaged with a high-content screening system using the Opera High-Content Imaging System (PerkinElmer). Cells were grown, reverse-transfected, and infected in Corning^®^ 384-Well Optical Imaging Flat Clear Bottom Black Polystyrene TC-Treated Microplates (Corning Inc.). Infections were allowed to proceed for 70 and 94 h before further processing. The cell media was removed from the cells by inverting the plate, and cells fixed with 4% formaldehyde in PBS. Cells were washed twice with 50 μl room temperature (RT) PBS (PBS removed by inverting the plate), permeabilized for 15 min at RT with 0.1% TritonX-100 in PBS (PBST), followed by inversion of the plate incubation in the dark for 45 min with AlexaFluor-647-phalloidin (1:50) (Invitrogen Molecular Probes; Cat. No. A22287) in PBS with 1% bovine serum albumin. Nuclei were stained with diamidino-2-phenylindole (1 mg/ml) in PBS. Cells were analyzed by automated microscopy using the Opera High-Content Imaging System (PerkinElmer) and a 20× Air lens.

## Author Contributions

WD and PM analyzed and wrote the paper. JJ, NY, PW, AM, and AA contributed to the study design and analysis. KK contributed to the metabolic pathway analysis. TF, JJ, and LP-M contributed to data analysis. MC helped with neonatal sample analysis, and KM, MA, and MC performed the GPCR RNAi screen. PG conceived and contributed to design, analysis, and writing the paper.

## Conflict of Interest Statement

The authors declare that the research was conducted in the absence of any commercial or financial relationships that could be construed as a potential conflict of interest.

## Footnotes

^1^http://www.innatedb.com

^2^http://www.string-db.org

## Supplementary Material

Table S1 Members of STRING networks.

Table S2 Cell marker lists.

Table S3 GPCR SMARTpool RNAi screen.

Table S4 DEGs between cord and adult DCs.

## References

[1] Britt W. Controversies in the natural history of congenital human cytomegalovirus infection: the paradox of infection and disease in offspring of women with immunity prior to pregnancy. Med Microbiol Immunol (2015) 204:263–71. 10.1007/s00430-015-0399-9 25764180

[2] Rawlinson WD Boppana SB Fowler KB Kimberlin DW Lazzarotto T Alain S Congenital cytomegalovirus infection in pregnancy and the neonate: consensus recommendations for prevention, diagnosis, and therapy. Lancet Infect Dis (2017) 17:e177–88. 10.1016/S1473-3099(17)30143-3 28291720

[3] Britt WJ. Congenital human cytomegalovirus infection and the enigma of maternal immunity. J Virol (2017) 91:e02392–16. 10.1128/JVI.02392-16 28490582 PMC5512250

[4] Vollmer B Seibold-Weiger K Schmitz-Salue C Hamprecht K Goelz R Krageloh-Mann I Postnatally acquired cytomegalovirus infection via breast milk: effects on hearing and development in preterm infants. Pediatr Infect Dis J (2004) 23:322–7. 10.1097/00006454-200404000-00009 15071286

[5] Adkins B Neonatal immunology: responses to pathogenic microorganisms and epigenetics reveal an “immunodiverse” developmental state. Immunol Res (2013) 57:246–57. 10.1007/s12026-013-8439-2 24214026

[6] PrabhuDas M Adkins B Gans H King C Levy O Ramilo O Challenges in infant immunity: implications for responses to infection and vaccines. Nat Immunol (2011) 12:189–94. 10.1038/ni0311-189 21321588

[7] Siefker DT Adkins B Rapid CD8(+) function is critical for protection of neonatal mice from an extracellular bacterial enteropathogen. Front Pediatr (2016) 4:141 10.3389/fped.2016.00141 28119902 PMC5220481

[8] Dickinson P Smith CL Forster T Craigon M Ross AJ Khondoker MR Whole blood gene expression profiling of neonates with confirmed bacterial sepsis. Genom Data (2015) 3:41–8. 10.1016/j.gdata.2014.11.003 26484146 PMC4535963

[9] Smith CL Dickinson P Forster T Craigon M Ross A Khondoker MR Identification of a human neonatal immune-metabolic network associated with bacterial infection. Nat Commun (2014) 5:4649. 10.1038/ncomms5649 25120092 PMC4143936

[10] McGovern N Shin A Low G Low D Duan K Yao LJ Human fetal dendritic cells promote prenatal T-cell immune suppression through arginase-2. Nature (2017) 546:662–6. 10.1038/nature22795 28614294 PMC6588541

[11] Ghazal P Dickinson P Smith CL. Early life response to infection. Curr Opin Infect Dis (2013) 26:213–8. 10.1097/QCO.0b013e32835fb8bf 23449137

[12] Mold JE Venkatasubrahmanyam S Burt TD Michaëlsson J Rivera JM Galkina SA Fetal and adult hematopoietic stem cells give rise to distinct T cell lineages in humans. Science (2010) 330:1695–9. 10.1126/science.1196509 21164017 PMC3276679

[13] Rhodes-Feuillette A Canivet M Champsaur H Gluckman E Mazeron MC Peries J. Circulating interferon in cytomegalovirus infected bone-marrow-transplant recipients and in infants with congenital cytomegalovirus disease. J Interferon Res (1983) 3:45–52. 10.1089/jir.1983.3.45 6188792

[14] Renneson J Dutta B Goriely S Danis B Lecomte S Laes JF IL-12 and type I IFN response of neonatal myeloid DC to human CMV infection. Eur J Immunol (2009) 39:2789–99. 10.1002/eji.200939414 19637227

[15] Martinon F Burns K Tschopp J. The inflammasome: a molecular platform triggering activation of inflammatory caspases and processing of proIL-beta. Mol Cell (2002) 10:417–26. 10.1016/S1097-2765(02)00599-3 12191486

[16] Heil F Hemmi H Hochrein H Ampenberger F Kirschning C Akira S Species-specific recognition of single-stranded RNA via toll-like receptor 7 and 8. Science (2004) 303:1526–9. 10.1126/science.1093620 14976262

[17] Smith PD Shimamura M Musgrove LC Dennis EA Bimczok D Novak L Cytomegalovirus enhances macrophage TLR expression and MyD88-mediated signal transduction to potentiate inducible inflammatory responses. J Immunol (2014) 193:5604–12. 10.4049/jimmunol.1302608 25355920 PMC4239158

[18] Zhang Y El-Far M Dupuy FP Abdel-Hakeem MS He Z Procopio FA HCV RNA activates APCs via TLR7/TLR8 while virus selectively stimulates macrophages without inducing antiviral responses. Sci Rep (2016) 6:29447. 10.1038/srep29447 27385120 PMC4935957

[19] Stewart CR Stuart LM Wilkinson K van Gils JM Deng J Halle A CD36 ligands promote sterile inflammation through assembly of a toll-like receptor 4 and 6 heterodimer. Nat Immunol (2010) 11:155–61. 10.1038/ni.1836 20037584 PMC2809046

[20] Fabregat A Sidiropoulos K Garapati P Gillespie M Hausmann K Haw R The reactome pathway knowledgebase. Nucleic Acids Res (2016) 44:D481–7. 10.1093/nar/gkv1351 26656494 PMC4702931

[21] Milacic M Haw R Rothfels K Wu G Croft D Hermjakob H Annotating cancer variants and anti-cancer therapeutics in reactome. Cancers (Basel) (2012) 4:1180–211. 10.3390/cancers4041180 24213504 PMC3712731

[22] Agbaga MP Brush RS Mandal MN Henry K Elliott MH Anderson RE. Role of stargardt-3 macular dystrophy protein (ELOVL4) in the biosynthesis of very long chain fatty acids. Proc Natl Acad Sci U S A (2008) 105:12843–8. 10.1073/pnas.0802607105 18728184 PMC2525561

[23] Moon YA Shah NA Mohapatra S Warrington JA Horton JD. Identification of a mammalian long chain fatty acyl elongase regulated by sterol regulatory element-binding proteins. J Biol Chem (2001) 276:45358–66. 10.1074/jbc.M108413200 11567032

[24] Tamura K Makino A Hullin-Matsuda F Kobayashi T Furihata M Chung S Novel lipogenic enzyme ELOVL7 is involved in prostate cancer growth through saturated long-chain fatty acid metabolism. Cancer Res (2009) 69:8133–40. 10.1158/0008-5472.CAN-09-0775 19826053

[25] Munger J Bennett BD Parikh A Feng XJ McArdle J Rabitz HA Systems-level metabolic flux profiling identifies fatty acid synthesis as a target for antiviral therapy. Nat Biotechnol (2008) 26:1179–86. 10.1038/nbt.1500 18820684 PMC2825756

[26] Purdy JG Shenk T Rabinowitz JD. Fatty acid elongase 7 catalyzes lipidome remodeling essential for human cytomegalovirus replication. Cell Rep (2015) 10:1375–85. 10.1016/j.celrep.2015.02.003 25732827 PMC4354725

[27] Koyuncu E Purdy JG Rabinowitz JD Shenk T. Saturated very long chain fatty acids are required for the production of infectious human cytomegalovirus progeny. PLoS Pathog (2013) 9:e1003333. 10.1371/journal.ppat.1003333 23696731 PMC3656100

[28] Sato T Yoshida Y Morita A Mori N Miura S. Glycerol-3-phosphate dehydrogenase 1 deficiency induces compensatory amino acid metabolism during fasting in mice. Metabolism (2016) 65:1646–56. 10.1016/j.metabol.2016.08.005 27733253

[29] Center DM Cruikshank W Modulation of lymphocyte migration by human lymphokines. I. Identification and characterization of chemoattractant activity for lymphocytes from mitogen-stimulated mononuclear cells. J Immunol (1982) 128:2563–8. 7042840

[30] Cruikshank WW Berman JS Theodore AC Bernardo J Center DM. Lymphokine activation of T4+ T lymphocytes and monocytes. J Immunol (1987) 138:3817–23. 3108375

[31] Garcia S Hartkamp LM Malvar-Fernandez B van Es IE Lin H Wong J Colony-stimulating factor (CSF) 1 receptor blockade reduces inflammation in human and murine models of rheumatoid arthritis. Arthritis Res Ther (2016) 18:75. 10.1186/s13075-016-0973-6 27036883 PMC4818474

[32] Kim JI Ho IC Grusby MJ Glimcher LH. The transcription factor c-Maf controls the production of interleukin-4 but not other Th2 cytokines. Immunity (1999) 10:745–51. 10.1016/S1074-7613(00)80073-4 10403649

[33] Russo I Bubacco L Greggio E. LRRK2 and neuroinflammation: partners in crime in Parkinson’s disease? J Neuroinflammation (2014) 11:52. 10.1186/1742-2094-11-52 24655756 PMC3994422

[34] Basso K Dalla-Favera R. Roles of BCL6 in normal and transformed germinal center B cells. Immunol Rev (2012) 247:172–83. 10.1111/j.1600-065X.2012.01112.x 22500840

[35] Chu DH Morita CT Weiss A. The Syk family of protein tyrosine kinases in T-cell activation and development. Immunol Rev (1998) 165:167–80. 10.1111/j.1600-065X.1998.tb01238.x 9850860

[36] Cornall RJ Cheng AM Pawson T Goodnow CC. Role of Syk in B-cell development and antigen-receptor signaling. Proc Natl Acad Sci U S A (2000) 97:1713–8. 10.1073/pnas.97.4.1713 10677523 PMC26501

[37] Dragone LL Myers MD White C Sosinowski T Weiss A. SRC-like adaptor protein regulates B cell development and function. J Immunol (2006) 176:335–45. 10.4049/jimmunol.176.1.335 16365426

[38] Latour S Fournel M Veillette A. Regulation of T-cell antigen receptor signalling by Syk tyrosine protein kinase. Mol Cell Biol (1997) 17:4434–41. 10.1128/MCB.17.8.4434 9234701 PMC232297

[39] Misra RS Shi G Moreno-Garcia ME Thankappan A Tighe M Mousseau B G alpha q-containing G proteins regulate B cell selection and survival and are required to prevent B cell-dependent autoimmunity. J Exp Med (2010) 207:1775–89. 10.1084/jem.20092735 20624888 PMC2916136

[40] Sosinowski T Pandey A Dixit VM Weiss A. Src-like adaptor protein (SLAP) is a negative regulator of T cell receptor signaling. J Exp Med (2000) 191:463–74. 10.1084/jem.191.3.463 10662792 PMC2195826

[41] Bailey CC Zhong G Huang IC Farzan M. IFITM-family proteins: the cell’s first line of antiviral defense. Annu Rev Virol (2014) 1:261–83. 10.1146/annurev-virology-031413-085537 25599080 PMC4295558

[42] Cheriyath V Leaman DW Borden EC. Emerging roles of FAM14 family members (G1P3/ISG 6–16 and ISG12/IFI27) in innate immunity and cancer. J Interferon Cytokine Res (2011) 31:173–81. 10.1089/jir.2010.0105 20939681 PMC6468951

[43] Choi UY Kang JS Hwang YS Kim YJ. Oligoadenylate synthase-like (OASL) proteins: dual functions and associations with diseases. Exp Mol Med (2015) 47:e144. 10.1038/emm.2014.110 25744296 PMC4351405

[44] Fensterl V Sen GC Interferon-induced Ifit proteins: their role in viral pathogenesis. J Virol (2015) 89:2462–8. 10.1128/JVI.02744-14 25428874 PMC4325746

[45] Imaizumi T Yano C Numata A Tsugawa K Hayakari R Matsumiya T Interferon (IFN)-induced protein 35 (IFI35), a type I interferon-dependent transcript, upregulates inflammatory signaling pathways by activating toll-like receptor 3 in human mesangial cells. Kidney Blood Press Res (2016) 41:635–42. 10.1159/000447932 27639618

[46] Verhelst J Hulpiau P Saelens X. Mx proteins: antiviral gatekeepers that restrain the uninvited. Microbiol Mol Biol Rev (2013) 77:551–66. 10.1128/MMBR.00024-13 24296571 PMC3973384

[47] Vestal DJ Jeyaratnam JA. The guanylate-binding proteins: emerging insights into the biochemical properties and functions of this family of large interferon-induced guanosine triphosphatase. J Interferon Cytokine Res (2011) 31:89–97. 10.1089/jir.2010.0102 21142871 PMC3021356

[48] Malakhov MP Malakhova OA Kim KI Ritchie KJ Zhang DE. UBP43 (USP18) specifically removes ISG15 from conjugated proteins. J Biol Chem (2002) 277:9976–81. 10.1074/jbc.M109078200 11788588

[49] Stremlau M Perron M Lee M Li Y Song B Javanbakht H Specific recognition and accelerated uncoating of retroviral capsids by the TRIM5alpha restriction factor. Proc Natl Acad Sci U S A (2006) 103:5514–9. 10.1073/pnas.0509996103 16540544 PMC1459386

[50] Mandell MA Jain A Arko-Mensah J Chauhan S Kimura T Dinkins C TRIM proteins regulate autophagy and can target autophagic substrates by direct recognition. Dev Cell (2014) 30:394–409. 10.1016/j.devcel.2014.06.013 25127057 PMC4146662

[51] O’Connor C Pertel T Gray S Robia SL Bakowska JC Luban J p62/sequestosome-1 associates with and sustains the expression of retroviral restriction factor TRIM5alpha. J Virol (2010) 84:5997–6006. 10.1128/JVI.02412-09 20357094 PMC2876647

[52] Lascano J Uchil PD Mothes W Luban J. TRIM5 retroviral restriction activity correlates with the ability to induce innate immune signaling. J Virol (2015) 90:308–16. 10.1128/JVI.02496-15 26468522 PMC4702541

[53] Rajsbaum R Versteeg GA Schmid S Maestre AM Belicha-Villanueva A Martínez-Romero C Unanchored K48-linked polyubiquitin synthesized by the E3-ubiquitin ligase TRIM6 stimulates the interferon-IKKε kinase-mediated antiviral response. Immunity (2014) 40:880–95. 10.1016/j.immuni.2014.04.018 24882218 PMC4114019

[54] Moreno-Fernandez ME Aliberti J Groeneweg S Köhl J Chougnet CA. A novel role for the receptor of the complement cleavage fragment C5a, C5aR1, in CCR5-mediated entry of HIV into macrophages. AIDS Res Hum Retroviruses (2016) 32:399–408. 10.1089/AID.2015.0099 26537334 PMC4817595

[55] Hodrea J Majai G Doró Z Zahuczky G Pap A Rajnavölgyi É The glucocorticoid dexamethasone programs human dendritic cells for enhanced phagocytosis of apoptotic neutrophils and inflammatory response. J Leukoc Biol (2012) 91:127–36. 10.1189/jlb.0511243 22028334

[56] Barlic J Andrews JD Kelvin AA Bosinger SE DeVries ME Xu L Regulation of tyrosine kinase activation and granule release through beta-arrestin by CXCRI. Nat Immunol (2000) 1:227–33. 10.1038/79767 10973280

[57] Barlic J Khandaker MH Mahon E Andrews J DeVries ME Mitchell GB Beta-arrestins regulate interleukin-8-induced CXCR1 internalization. J Biol Chem (1999) 274:16287–94. 10.1074/jbc.274.23.16287 10347185

[58] Arnolds KL Lares AP Spencer JV. The US27 gene product of human cytomegalovirus enhances signaling of host chemokine receptor CXCR4. Virology (2013) 439:122–31. 10.1016/j.virol.2013.02.006 23490053 PMC3639318

[59] Tadagaki K Tudor D Gbahou F Tschische P Waldhoer M Bomsel M Human cytomegalovirus-encoded UL33 and UL78 heteromerize with host CCR5 and CXCR4 impairing their HIV coreceptor activity. Blood (2012) 119:4908–18. 10.1182/blood-2011-08-372516 22496149

[60] Imai T Baba M Nishimura M Kakizaki M Takagi S Yoshie O. The T cell-directed CC chemokine TARC is a highly specific biological ligand for CC chemokine receptor 4. J Biol Chem (1997) 272:15036–42. 10.1074/jbc.272.23.15036 9169480

[61] Imai T Yoshida T Baba M Nishimura M Kakizaki M Yoshie O. Molecular cloning of a novel T cell-directed CC chemokine expressed in thymus by signal sequence trap using Epstein-Barr virus vector. J Biol Chem (1996) 271:21514–21. 10.1074/jbc.271.35.21514 8702936

[62] Kang S Xie J Ma S Liao W Zhang J Luo R. Targeted knock down of CCL22 and CCL17 by siRNA during DC differentiation and maturation affects the recruitment of T subsets. Immunobiology (2010) 215:153–62. 10.1016/j.imbio.2009.03.001 19450895

[63] Mishalian I Bayuh R Eruslanov E Michaeli J Levy L Zolotarov L Neutrophils recruit regulatory T-cells into tumors via secretion of CCL17 – a new mechanism of impaired antitumor immunity. Int J Cancer (2014) 135:1178–86. 10.1002/ijc.28770 24501019

[64] Riezu-Boj JI Larrea E Aldabe R Guembe L Casares N Galeano E Hepatitis C virus induces the expression of CCL17 and CCL22 chemokines that attract regulatory T cells to the site of infection. J Hepatol (2011) 54:422–31. 10.1016/j.jhep.2010.07.014 21129807

[65] Venkataraman C Kuo F. The G-protein coupled receptor, GPR84 regulates IL-4 production by T lymphocytes in response to CD3 crosslinking. Immunol Lett (2005) 101:144–53. 10.1016/j.imlet.2005.05.010 15993493

[66] Gouwy M Struyf S Leutenez L Pörtner N Sozzani S Van Damme J Chemokines and other GPCR ligands synergize in receptor-mediated migration of monocyte-derived immature and mature dendritic cells. Immunobiology (2014) 219:218–29. 10.1016/j.imbio.2013.10.004 24268109

[67] Groot-Kormelink PJ Fawcett L Wright PD Gosling M Kent TC. Quantitative GPCR and ion channel transcriptomics in primary alveolar macrophages and macrophage surrogates. BMC Immunol (2012) 13:57. 10.1186/1471-2172-13-57 23102269 PMC3542584

[68] Lattin J Zidar DA Schroder K Kellie S Hume DA Sweet MJ. G-protein-coupled receptor expression, function, and signaling in macrophages. J Leukoc Biol (2007) 82:16–32. 10.1189/jlb.0107051 17456803

[69] Lattin JE Schroder K Su AI Walker JR Zhang J Wiltshire T Expression analysis of G protein-coupled receptors in mouse macrophages. Immunome Res (2008) 4:5. 10.1186/1745-7580-4-5 18442421 PMC2394514

[70] Shi GX Harrison K Han SB Moratz C Kehrl JH. Toll-like receptor signaling alters the expression of regulator of G protein signaling proteins in dendritic cells: implications for G protein-coupled receptor signaling. J Immunol (2004) 172:5175–84. 10.4049/jimmunol.172.9.5175 15100254

[71] Idzko M Dichmann S Ferrari D Di Virgilio F la Sala A Girolomoni G Nucleotides induce chemotaxis and actin polymerization in immature but not mature human dendritic cells via activation of pertussis toxin-sensitive P2y receptors. Blood (2002) 100:925–32. 10.1182/blood.V100.3.925 12130504

[72] Idzko M la Sala A Ferrari D Panther E Herouy Y Dichmann S Expression and function of histamine receptors in human monocyte-derived dendritic cells. J Allergy Clin Immunol (2002) 109:839–46. 10.1067/mai.2002.124044 11994709

[73] Panther E Idzko M Corinti S Ferrari D Herouy Y Mockenhaupt M The influence of lysophosphatidic acid on the functions of human dendritic cells. J Immunol (2002) 169:4129–35. 10.4049/jimmunol.169.8.4129 12370341

[74] Idzko M Panther E Corinti S Morelli A Ferrari D Herouy Y Sphingosine 1-phosphate induces chemotaxis of immature and modulates cytokine-release in mature human dendritic cells for emergence of Th2 immune responses. FASEB J (2002) 16:625–7. 10.1096/fj.01-0625fje 11919175

[75] Liebscher I Müller U Teupser D Engemaier E Engel KM Ritscher L Altered immune response in mice deficient for the G protein-coupled receptor GPR34. J Biol Chem (2011) 286:2101–10. 10.1074/jbc.M110.196659 21097509 PMC3023507

[76] Deng W Wang X Xiao J Chen K Zhou H Shen D Loss of regulator of G protein signaling 5 exacerbates obesity, hepatic steatosis, inflammation and insulin resistance. PLoS One (2012) 7:e30256 10.1371/journal.pone.0030256 22272317 PMC3260252

[77] Han Y Wu Y Yang C Huang J Guo Y Liu L Dynamic and specific immune responses against multiple tumor antigens were elicited in patients with hepatocellular carcinoma after cell-based immunotherapy. J Transl Med (2017) 15:64. 10.1186/s12967-017-1165-0 28330473 PMC5363021

[78] Serbina NV Jia T Hohl TM Pamer EG. Monocyte-mediated defense against microbial pathogens. Annu Rev Immunol (2008) 26:421–52. 10.1146/annurev.immunol.26.021607.090326 18303997 PMC2921669

[79] Xue D Lu M Gao B Qiao X Zhang Y. Screening for transcription factors and their regulatory small molecules involved in regulating the functions of CL1-5 cancer cells under the effects of macrophage-conditioned medium. Oncol Rep (2014) 31:1323–33. 10.3892/or.2013.2937 24366584

[80] Stäubert C Broom OJ Nordström A. Hydroxycarboxylic acid receptors are essential for breast cancer cells to control their lipid/fatty acid metabolism. Oncotarget (2015) 6:19706–20. 10.18632/oncotarget.3565 25839160 PMC4637315

[81] Das A Dinh PX Panda D Pattnaik AK. Interferon-inducible protein IFI35 negatively regulates RIG-I antiviral signaling and supports vesicular stomatitis virus replication. J Virol (2014) 88:3103–13. 10.1128/JVI.03202-13 24371060 PMC3957919

[82] Saito T Hirai R Loo YM Owen D Johnson CL Sinha SC Regulation of innate antiviral defenses through a shared repressor domain in RIG-I and LGP2. Proc Natl Acad Sci U S A (2007) 104:582–7. 10.1073/pnas.0606699104 17190814 PMC1766428

[83] Zhao C Denison C Huibregtse JM Gygi S Krug RM. Human ISG15 conjugation targets both IFN-induced and constitutively expressed proteins functioning in diverse cellular pathways. Proc Natl Acad Sci U S A (2005) 102:10200–5. 10.1073/pnas.0504754102 16009940 PMC1177427

[84] Johnson CL Gale M. CARD games between virus and host get a new player. Trends Immunol (2006) 27:1–4. 10.1016/j.it.2005.11.004 16309964

[85] Childs KS Randall RE Goodbourn S. LGP2 plays a critical role in sensitizing mda-5 to activation by double-stranded RNA. PLoS One (2013) 8:e64202. 10.1371/journal.pone.0064202 23671710 PMC3650065

[86] Reikine S Nguyen JB Modis Y. Pattern recognition and signaling mechanisms of RIG-I and MDA5. Front Immunol (2014) 5:342. 10.3389/fimmu.2014.00342 25101084 PMC4107945

[87] Rothenfusser S Goutagny N DiPerna G Gong M Monks BG Schoenemeyer A The RNA helicase Lgp2 inhibits TLR-independent sensing of viral replication by retinoic acid-inducible gene-I. J Immunol (2005) 175:5260–8. 10.4049/jimmunol.175.8.5260 16210631

[88] Childs K Randall R Goodbourn S. Paramyxovirus V proteins interact with the RNA Helicase LGP2 to inhibit RIG-I-dependent interferon induction. J Virol (2012) 86:3411–21. 10.1128/JVI.06405-11 22301134 PMC3302505

[89] Komuro A Horvath CM. RNA- and virus-independent inhibition of antiviral signaling by RNA helicase LGP2. J Virol (2006) 80:12332–42. 10.1128/JVI.01325-06 17020950 PMC1676302

[90] Hsiang TY Zhao C Krug RM. Interferon-induced ISG15 conjugation inhibits influenza A virus gene expression and replication in human cells. J Virol (2009) 83:5971–7. 10.1128/JVI.01667-08 19357168 PMC2687383

[91] Okumura A Lu G Pitha-Rowe I Pitha PM. Innate antiviral response targets HIV-1 release by the induction of ubiquitin-like protein ISG15. Proc Natl Acad Sci U S A (2006) 103:1440–5. 10.1073/pnas.0510518103 16434471 PMC1360585

[92] Okumura A Pitha PM Harty RN ISG15 inhibits Ebola VP40 VLP budding in an L-domain-dependent manner by blocking Nedd4 ligase activity. Proc Natl Acad Sci U S A (2008) 105:3974–9. 10.1073/pnas.0710629105 18305167 PMC2268823

[93] Broering R Zhang X Kottilil S Trippler M Jiang M Lu M The interferon stimulated gene 15 functions as a proviral factor for the hepatitis C virus and as a regulator of the IFN response. Gut (2010) 59:1111–9. 10.1136/gut.2009.195545 20639253

[94] Jeon YJ Choi JS Lee JY Yu KR Kim SM Ka SH ISG15 modification of filamin B negatively regulates the type I interferon-induced JNK signalling pathway. EMBO Rep (2009) 10:374–80. 10.1038/embor.2009.23 19270716 PMC2672892

[95] Knight E Cordova B. IFN-induced 15-kDa protein is released from human lymphocytes and monocytes. J Immunol (1991) 146:2280–4. 2005397

[96] Loeb KR Haas AL. The interferon-inducible 15-kDa ubiquitin homolog conjugates to intracellular proteins. J Biol Chem (1992) 267:7806–13. 1373138

[97] Okumura F Okumura AJ Uematsu K Hatakeyama S Zhang DE Kamura T. Activation of double-stranded RNA-activated protein kinase (PKR) by interferon-stimulated gene 15 (ISG15) modification down-regulates protein translation. J Biol Chem (2013) 288:2839–47. 10.1074/jbc.M112.401851 23229543 PMC3554948

[98] Shi HX Yang K Liu X Liu XY Wei B Shan YF Positive regulation of interferon regulatory factor 3 activation by Herc5 via ISG15 modification. Mol Cell Biol (2010) 30:2424–36. 10.1128/MCB.01466-09 20308324 PMC2863703

[99] Zou W Kim JH Handidu A Li X Kim KI Yan M Microarray analysis reveals that type I interferon strongly increases the expression of immune-response related genes in Ubp43 (Usp18) deficient macrophages. Biochem Biophys Res Commun (2007) 356:193–9. 10.1016/j.bbrc.2007.02.101 17349616 PMC1868545

[100] Burkart C Fan JB Zhang DE. Two independent mechanisms promote expression of an N-terminal truncated USP18 isoform with higher DeISGylation activity in the nucleus. J Biol Chem (2012) 287:4883–93. 10.1074/jbc.M111.255570 22170061 PMC3281662

[101] Hu Y Wang J Yang B Zheng N Qin M Ji Y Guanylate binding protein 4 negatively regulates virus-induced type I IFN and antiviral response by targeting IFN regulatory factor 7. J Immunol (2011) 187:6456–62. 10.4049/jimmunol.1003691 22095711

[102] Lee MS Kim B Oh GT Kim YJ. OASL1 inhibits translation of the type I interferon-regulating transcription factor IRF7. Nat Immunol (2013) 14:346–55. 10.1038/ni.2535 23416614

[103] Young JA Sermwittayawong D Kim HJ Nandu S An N Erdjument-Bromage H Fas-associated death domain (FADD) and the E3 ubiquitin-protein ligase TRIM21 interact to negatively regulate virus-induced interferon production. J Biol Chem (2011) 286:6521–31. 10.1074/jbc.M110.172288 21183682 PMC3057824

[104] Zhu J Zhang Y Ghosh A Cuevas RA Forero A Dhar J Antiviral activity of human OASL protein is mediated by enhancing signaling of the RIG-I RNA sensor. Immunity (2014) 40:936–48. 10.1016/j.immuni.2014.05.007 24931123 PMC4101812

[105] Manocha GD Mishra R Sharma N Kumawat KL Basu A Singh SK. Regulatory role of TRIM21 in the type-I interferon pathway in Japanese encephalitis virus-infected human microglial cells. J Neuroinflammation (2014) 11:24. 10.1186/1742-2094-11-24 24485101 PMC3922089

[106] Barnes BJ Richards J Mancl M Hanash S Beretta L Pitha PM. Global and distinct targets of IRF-5 and IRF-7 during innate response to viral infection. J Biol Chem (2004) 279:45194–207. 10.1074/jbc.M400726200 15308637

[107] Ben-Zvi T Yayon A Gertler A Monsonego-Ornan E. Suppressors of cytokine signaling (SOCS) 1 and SOCS3 interact with and modulate fibroblast growth factor receptor signaling. J Cell Sci (2006) 119:380–7. 10.1242/jcs.02740 16410555

[108] Masih-Khan E Trudel S Heise C Li Z Paterson J Nadeem V MIP-1alpha (CCL3) is a downstream target of FGFR3 and RAS-MAPK signaling in multiple myeloma. Blood (2006) 108:3465–71. 10.1182/blood-2006-04-017087 16849642

[109] Takeichi M. Dynamic contacts: rearranging adherens junctions to drive epithelial remodelling. Nat Rev Mol Cell Biol (2014) 15:397–410. 10.1038/nrm3802 24824068

[110] Hasegawa S Kobayashi H Kumagai M Nishimaru H Tarusawa E Kanda H Clustered protocadherins are required for building functional neural circuits. Front Mol Neurosci (2017) 10:114. 10.3389/fnmol.2017.00114 28484370 PMC5401904

[111] Keeler AB Molumby MJ Weiner JA. Protocadherins branch out: multiple roles in dendrite development. Cell Adh Migr (2015) 9:214–26. 10.1080/19336918.2014.1000069 25869446 PMC4594470

[112] Weiner JA Jontes JD Protocadherins, not prototypical: a complex tale of their interactions, expression, and functions. Front Mol Neurosci (2013) 6:4 10.3389/fnmol.2013.00004 23515683 PMC3601302

[113] Shan M Su Y Kang W Gao R Li X Zhang G. Aberrant expression and functions of protocadherins in human malignant tumors. Tumour Biol (2016) 37:12969–81. 10.1007/s13277-016-5169-9 27449047

[114] Hirabayashi T Yagi T. Protocadherins in neurological diseases. Adv Neurobiol (2014) 8:293–314. 10.1007/978-1-4614-8090-7_13 25300142

[115] Bray NJ Kirov G Owen RJ Jacobsen NJ Georgieva L Williams HJ Screening the human protocadherin 8 (PCDH8) gene in schizophrenia. Genes Brain Behav (2002) 1:187–91. 10.1034/j.1601-183X.2002.10307.x 12884975

[116] Yamagata K Andreasson KI Sugiura H Maru E Dominique M Irie Y Arcadlin is a neural activity-regulated cadherin involved in long term potentiation. J Biol Chem (1999) 274:19473–11979. 10.1074/jbc.274.27.19473 10383464

[117] Marshall CR Noor A Vincent JB Lionel AC Feuk L Skaug J Structural variation of chromosomes in autism spectrum disorder. Am J Hum Genet (2008) 82:477–88. 10.1016/j.ajhg.2007.12.009 18252227 PMC2426913

[118] Huang YT Heist RS Chirieac LR Lin X Skaug V Zienolddiny S Genome-wide analysis of survival in early-stage non-small-cell lung cancer. J Clin Oncol (2009) 27:2660–7. 10.1200/JCO.2008.18.7906 19414679 PMC2690391

[119] Leshchenko VV Kuo PY Shaknovich R Yang DT Gellen T Petrich A Genomewide DNA methylation analysis reveals novel targets for drug development in mantle cell lymphoma. Blood (2010) 116:1025–34. 10.1182/blood-2009-12-257485 20427703 PMC2938124

[120] Yu JS Koujak S Nagase S Li CM Su T Wang X PCDH8, the human homolog of PAPC, is a candidate tumor suppressor of breast cancer. Oncogene (2008) 27:4657–65. 10.1038/onc.2008.101 18408767 PMC3013056

[121] Chen WV Maniatis T. Clustered protocadherins. Development (2013) 140:3297–302. 10.1242/dev.090621 23900538 PMC3737714

[122] Hasegawa S Hamada S Kumode Y Esumi S Katori S Fukuda E The protocadherin-alpha family is involved in axonal coalescence of olfactory sensory neurons into glomeruli of the olfactory bulb in mouse. Mol Cell Neurosci (2008) 38:66–79. 10.1016/j.mcn.2008.01.016 18353676

[123] Katori S Hamada S Noguchi Y Fukuda E Yamamoto T Yamamoto H Protocadherin-alpha family is required for serotonergic projections to appropriately innervate target brain areas. J Neurosci (2009) 29:9137–47. 10.1523/JNEUROSCI.5478-08.2009 19625505 PMC6665563

[124] Miyake K Hirasawa T Soutome M Itoh M Goto Y Endoh K The protocadherins, PCDHB1 and PCDH7, are regulated by MeCP2 in neuronal cells and brain tissues: implication for pathogenesis of Rett syndrome. BMC Neurosci (2011) 12:81. 10.1186/1471-2202-12-81 21824415 PMC3160964

[125] O’Roak BJ Vives L Girirajan S Karakoc E Krumm N Coe BP Sporadic autism exomes reveal a highly interconnected protein network of de novo mutations. Nature (2012) 485:246–50. 10.1038/nature10989 22495309 PMC3350576

[126] Kadowaki N Liu YJ. Natural type I interferon-producing cells as a link between innate and adaptive immunity. Hum Immunol (2002) 63:1126–32. 10.1016/S0198-8859(02)00751-6 12480256

[127] Lalancette-Hébert M Faustino J Thammisetty SS Chip S Vexler ZS Kriz J Live imaging of the innate immune response in neonates reveals differential TLR2 dependent activation patterns in sterile inflammation and infection. Brain Behav Immun (2017) 65:312–27. 10.1016/j.bbi.2017.05.020 28579520 PMC6151183

[128] Vadillo E Dorantes-Acosta E Arriaga-Pizano L Chavez-Gonzalez A Reyes-Maldonado E Garrett KP Adult, but not neonatal, human lymphoid progenitors respond to TLR9 ligation by producing functional NK-like cells. Exp Hematol (2014) 42:562–73.e3. 10.1016/j.exphem.2014.03.008 24721609

[129] Mathias B Mira JC Rehfuss JP Rincon JC Ungaro R Nacionales DC LPS stimulation of cord blood reveals a newborn-specific neutrophil transcriptomic response and cytokine production. Shock (2017) 47:606–14. 10.1097/SHK.0000000000000800 28410545 PMC5407294

[130] Dela Peña-Ponce MG Rodriguez-Nieves J Bernhardt J Tuck R Choudhary N Mengual M Increasing JAK/STAT signaling function of infant CD4(+) T cells during the first year of life. Front Pediatr (2017) 5:15 10.3389/fped.2017.00015 28271056 PMC5318443

[131] Fike AJ Nguyen LT Kumova OK Carey AJ. Characterization of CD31 expression on murine and human neonatal T lymphocytes during development and activation. Pediatr Res (2017) 82:133–40. 10.1038/pr.2017.81 28355204 PMC5509503

[132] Köstlin N Vogelmann M Spring B Schwarz J Feucht J Härtel C Granulocytic myeloid-derived suppressor cells from human cord blood modulate T-helper cell response towards an anti-in ammatory phenotype. Immunology (2017) 152:89–101. 10.1111/imm.12751 28464218 PMC5543463

[133] Orlikowsky TW Dannecker GE Spring B Eichner M Hoffmann MK Poets CF. Effect of dexamethasone on B7 regulation and T cell activation in neonates and adults. Pediatr Res (2005) 57:656–61. 10.1203/01.PDR.0000156211.48307.F5 15718366

[134] Edgar R Domrachev M Lash AE. Gene expression omnibus: NCBI gene expression and hybridization array data repository. Nucleic Acids Res (2002) 30:207–10. 10.1093/nar/30.1.207 11752295 PMC99122

[135] R_Core_Team. R: A Language and Environment for Statistical Computing. R Foundation for Statistical Computing (2016). Available from: http://www.R-project.org

[136] Huber W Carey VJ Gentleman R Anders S Carlson M Carvalho BS Orchestrating high-throughput genomic analysis with Bioconductor. Nat Methods (2015) 12:115–21. 10.1038/nmeth.3252 25633503 PMC4509590

[137] Kauffmann A Gentleman R Huber W arrayQualityMetrics – a bioconductor package for quality assessment of microarray data. Bioinformatics (2009) 25:415–6. 10.1093/bioinformatics/btn647 19106121 PMC2639074

[138] Bolstad BM Irizarry RA Astrand M Speed TP. A comparison of normalization methods for high density oligonucleotide array data based on variance and bias. Bioinformatics (2003) 19:185–93. 10.1093/bioinformatics/19.2.185 12538238

[139] Ritchie ME Phipson B Wu D Hu Y Law CW Shi W limma powers differential expression analyses for RNA-sequencing and microarray studies. Nucleic Acids Res (2015) 43:e47. 10.1093/nar/gkv007 25605792 PMC4402510

[140] Holm S A simple sequentially rejective multiple test procedure. Scand J Stat (1979) 6:65–70.

[141] Porritt RA Hertzog PJ. Dynamic control of type I IFN signalling by an integrated network of negative regulators. Trends Immunol (2015) 36:150–60. 10.1016/j.it.2015.02.002 25725583

[142] Richards KH Macdonald A. Putting the brakes on the anti-viral response: negative regulators of type I interferon (IFN) production. Microbes Infect (2011) 13:291–302. 10.1016/j.micinf.2010.12.007 21256242

[143] Breuer K Foroushani AK Laird MR Chen C Sribnaia A Lo R InnateDB: systems biology of innate immunity and beyond – recent updates and continuing curation. Nucleic Acids Res (2013) 41:D1228–33. 10.1093/nar/gks1147 23180781 PMC3531080

[144] Lynn DJ Chan C Naseer M Yau M Lo R Sribnaia A Curating the innate immunity interactome. BMC Syst Biol (2010) 4:117. 10.1186/1752-0509-4-117 20727158 PMC2936296

[145] Lynn DJ Winsor GL Chan C Richard N Laird MR Barsky A InnateDB: facilitating systems-level analyses of the mammalian innate immune response. Mol Syst Biol (2008) 4:218. 10.1038/msb.2008.55 18766178 PMC2564732

[146] Szklarczyk D Franceschini A Wyder S Forslund K Heller D Huerta-Cepas J STRING v10: protein-protein interaction networks, integrated over the tree of life. Nucleic Acids Res (2015) 43:D447–52. 10.1093/nar/gku1003 25352553 PMC4383874

[147] Gustems M Borst E Benedict CA Pérez C Messerle M Ghazal P Regulation of the transcription and replication cycle of human cytomegalovirus is insensitive to genetic elimination of the cognate NF-kappaB binding sites in the enhancer. J Virol (2006) 80:9899–904. 10.1128/JVI.00640-06 16973595 PMC1617225

[148] Breitling R Armengaud P Amtmann A Herzyk P. Rank products: a simple, yet powerful, new method to detect differentially regulated genes in replicated microarray experiments. FEBS Lett (2004) 573:83–92. 10.1016/j.febslet.2004.07.055 15327980

